# Molecular Mechanisms Implicated in Myogenic Differentiation of Human Alveolar Mucosa-Derived Cells

**DOI:** 10.3390/ijms27198712

**Published:** 2026-09-29

**Authors:** Maksim Sorokin, Anton Buzdin, Anastasia Guryanova, Alexander Gudkov, Alexander Modestov, Maria Suntsova, Ilya Eremin, Andrey Pulin, Nastasia Kosheleva, Alla Zorina, Vadim Zorin, Dmitry Kudlay, Sergei Boichuk, Maria Vasileva, Maria Fedorova, Svetlana Vinokurova, Pavel Kopnin

**Affiliations:** 1 Institute of Personalized Oncology, I.M. Sechenov First Moscow State Medical University, Moscow 119991, Russia; sorokin_m_i@staff.sechenov.ru (M.S.);; 2 Omicsway Corp., 340 S Lemon Ave, 6040, Walnut, CA 91789, USA; 3 Oncobox Ltd., Moscow 121205, Russia; 4 Sirius University of Science and Technology, 1 Olimpiyskiy Ave, Sirius 354340, Krasnodar Region, Russia; 5 Petrovsky National Research Center of Surgery, Moscow 119991, Russia; 6 Pirogov National Medical and Surgical Center, Moscow 105203, Russia; 7 Institute for Regenerative Medicine, I.M. Sechenov First Moscow State Medical University, Moscow 119991, Russia; 8 Artgen Biotech, Moscow 119333, Russia; 9 Skincell LLC, Moscow 119333, Russia; 10 Department of Pharmacology, I.M. Sechenov First Moscow State Medical University, Moscow 119991, Russia; 11 Department of Pharmacognosy and Industrial Pharmacy, Lomonosov Moscow State University, Moscow 119992, Russia; 12 Department of Pathology, Kazan State Medical University, Kazan 420012, Russia; sergei.boichuk@kazangmu.ru; 13 Department of Radiotherapy and Radiology, Russian Medical Academy of Continuous Professional Education, Moscow 119454, Russia; 14 N.N. Blokhin National Medical Research Center of Oncology, Moscow 115522, Russiam.d.fedorova@ronc.ru (M.F.); s.vinokurova@ronc.ru (S.V.)

**Keywords:** myogenesis, alveolar mucosa mesenchymal cell, skeletal muscle, myogenic regulatory factors, transcriptional regulation network, gene expression, intracellular molecular pathways

## Abstract

Different approaches to skeletal muscle regeneration using progenitor cells are extensively studied as strategies for the treatment of muscle tissue pathologies. Recently, it was discovered that anatomically localized alveolar mucosa multipotent mesenchymal stromal cells (AMCs) are characterized by myogenic potential and high feasibility for muscle tissue recovery and regeneration. Although the resulting multinuclear myotubes express skeletal muscle-specific markers (skeletal myosin, actin, myogenin, and MyoD1), the exact molecular mechanism controlling myogenic differentiation of AMCs is still unclear and poorly scrutinized. In this research, we used a combination of large-scale transcriptome analysis and a bioinformatics approach to investigate molecular pathways and crucial nodes responsible for myogenic differentiation. We studied differentiation of AMC cells in 2D and 3D culture conditions and found core genes with significant expression changes during differentiation and compared them with skeletal muscle-derived stromal cells (SMCs). It appeared that differentiation of AMCs in 3D is significantly different from that in 2D. Moreover, differentiation of AMCs in 2D is closer to differentiation of SMCs in 2D than to AMCs in 3D. Unique properties of AMC differentiation in 3D may be attributed to a significant inhibition of the component of the PDGFRβ signaling pathway responsible for ruffle organization.

## 1. Introduction

Skeletal muscle is a major tissue of the human body that constitutes up to 40% of body weight [1]. Maintenance of the skeletal musculature in a functional state is crucial for the high quality of life and longevity. At the same time muscle tissue is subjected to the development of several serious dysfunctions, including muscular dystrophy, neuromuscular diseases, sarcopenia, cachexia (including cachexia related to cancer), and other congenital and acquired myopathies [2]. Some of these pathologies (such as muscular dystrophy) are incurable and cause disability or even death [3]. Traumatic injuries of the muscle tissue are also common, including surgical interventions and the failure of muscle sphincters of internal organs, including initial pathological changes and injuries resulting from surgical treatment. There are no effective treatment methods for these pathologies, even though physiological processes of muscle tissue are now well understood. Cell transplantation approaches involving cells with myogenic potential are considered promising, as they not only stimulate regeneration but also can improve undesirable processes occurring in altered muscle tissue, such as uncontrolled inflammation and fibrosis [4,5,6]. The ideal approach to the treatment of human muscle tissue diseases could be the transplantation of genetically modified autologous satellite cells [7]. However, it was shown that the expansion of satellite cells in vitro significantly reduces their myogenic potential in vivo, thus making their transplantation ineffective [8]. The approaches to stimulation of skeletal muscle regeneration based on different types of stem and progenitor cells are being studied intensively [9,10,11]. Currently, multipotent mesenchymal stromal cells (MMSCs) isolated from bone marrow, adipose tissue and umbilical cord blood, mononuclear cells and hematopoietic bone marrow-derived stem cells, mesoangioblasts, and myoblasts are considered as candidates for the recovery of muscle tissue.

Recently the ability of human gingiva-derived MMSCs to undergo myogenic differentiation was discovered [12]. We also found that the anatomical localization of gingiva segment as a cell source predetermines the myogenic potential of MMSCs. It was the alveolar mucosa-derived MMSC (AMC) population, but not attached gingiva-derived MMSCs (AGCs—cells obtained from attached gingiva), that contained a subpopulation of undifferentiated proliferation-competent cells able to form multinuclear myotubes expressing striated muscle markers: skeletal myosin and actin, myogenin and MyoD1 [12]. However, molecular mechanisms responsible for the myogenic differentiation of AMCs remained unexplored.

Here we used high-throughput transcriptomic profiling by RNA sequencing to elucidate molecular changes accompanying myogenic differentiation of AMCs in 2D and 3D culturing conditions.

## 2. Results

### 2.1. Anatomical Localization of Gingival Segments Dictates Myogenic Potential: Evaluation of Skeletal Muscle Markers in Differentiated AMCs and AGCs

**Figure 1:**
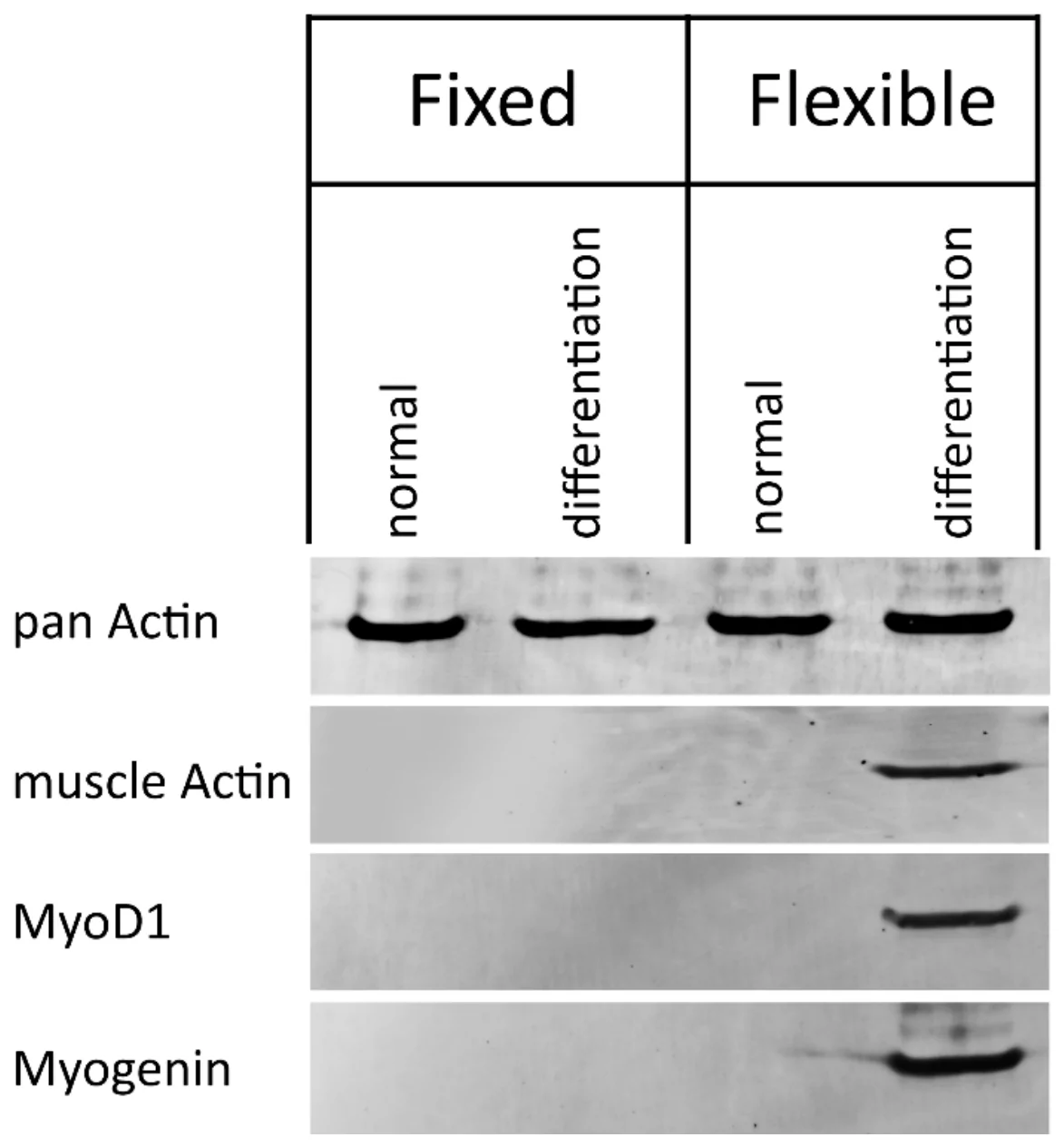
Western blot of proteins extracted from non-differentiated and differentiated AGCs (fixed—attached gingiva-derived MMSC) and AMCs (flexible—alveolar mucosa-derived MMSC) probed with anti-pan actin, anti-muscle actin, anti-MyoD1 and anti-myogenin antibodies. Pan-actin was used as a loading control.

**Figure 2:**
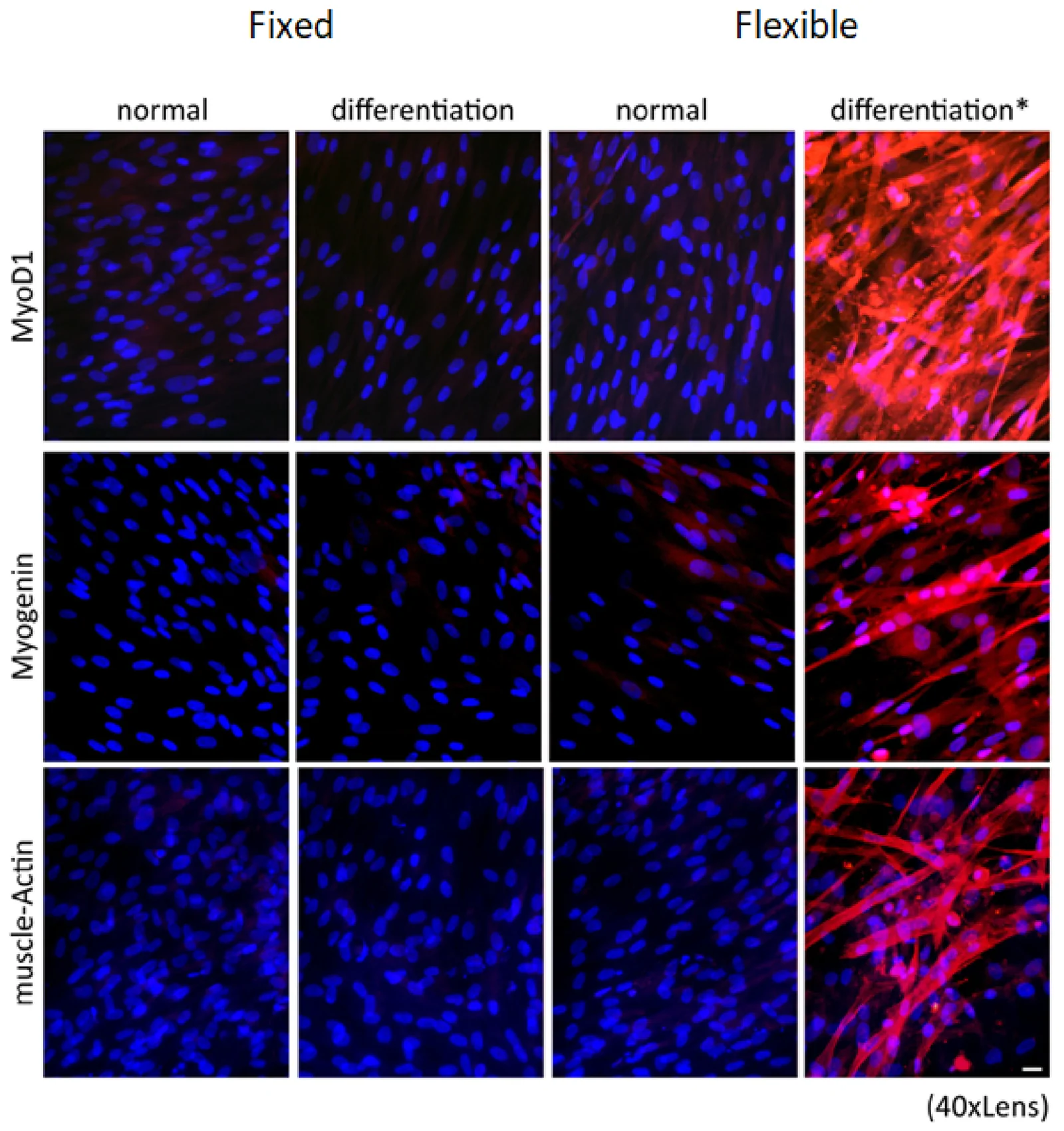
Immunofluorescence imaging of non-differentiated and differentiated AGCs (fixed—attached gingiva-derived MMSCs) and AMCs (flexible—alveolar mucosa-derived MMSCs), bar 10 μm. *—successful differentiation.

**Figure 3:**
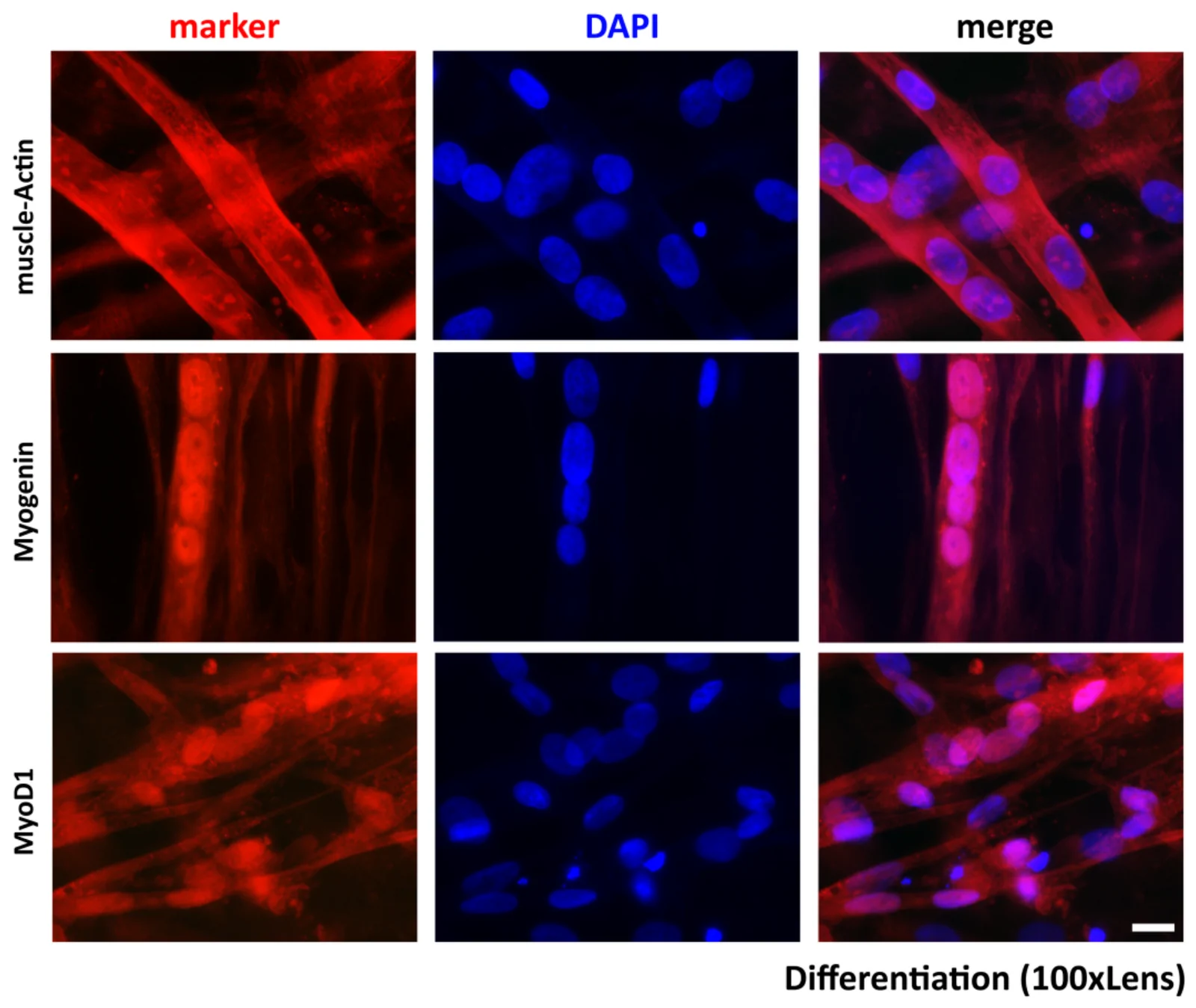
Enlarged (100× Lens) immunofluorescence imaging of differentiated AMCs (flexible—alveolar mucosa-derived MMSC) cells. Red dye—marker staining; blue—DAPI staining (bar 10 μm).

Anatomical localization of the gingival segment as a cell source predetermines the myogenic potential of MMSCs. It was previously demonstrated that alveolar mucosa-derived MMSC (AMC, flexible gingiva) population, but not attached gingiva-derived MMSCs (AGC, fixed gingiva) that contained a subpopulation of undifferentiated proliferation-competent cells, were able to form multinuclear myotubes expressing striated muscle biomarkers [12]. We present the validation of quantification of three selected skeletal muscle markers with Western blots (Figure 1) and immunofluorescence imaging (IF) (Figure 2). The list of marker proteins included muscle actin and the transcription factors myogenin and MyoD1. Western blot analysis and IF of AMCs and AGCs in non-differentiated (normal) and differentiated states revealed that the above-mentioned markers were expressed only in differentiated AMCs. For MyoD1 and myogenin proteins, nuclear localization in AMCs was clearly observed (Figure 3). We, therefore, focused our further research on AMCs and investigated gene expression changes during their myogenic differentiation.

To assess the myogenic differentiation potential of AMC and SMC cells, a selective analysis was carried out using fluorescence microscopy. Under 2D conditions, the percentage of areas covered by α-skeletal muscle actin-positive myotubes was calculated (Supplementary Figure S1A), whereas confocal microscopy was used to demonstrate 3D myogenic differentiation of the same cells (Supplementary Figure S1B).

### 2.2. Gene Expression Changes in 2D and 3D Conditions During AMC and SMC Myogenic Differentiation

Using RNA sequencing, we obtained the normalized gene expression levels for 38 AMC and (SMC) samples in two culturing conditions (2D and 3D spheroids) and differentiated and non-differentiated states (Table 1). The gene expression data were then processed using the Oncobox algorithm [13] to calculate pathway activation levels (PALs). In total, we analyzed the expression of 36,596 genes and the activation status of 3044 intracellular molecular pathways.

**Figure 4:**
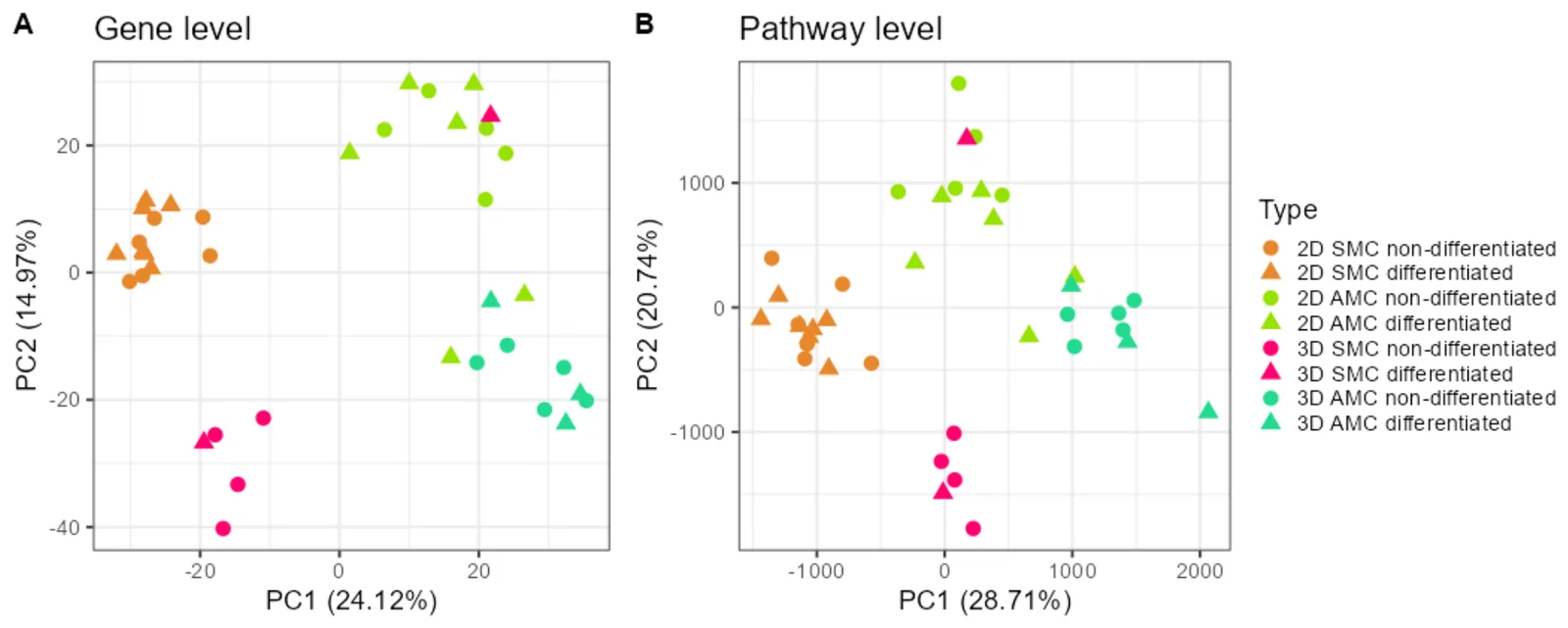
Principal component analysis (PCA) of RNAseq gene expression of differentiated and non-differentiated alveolar mucosa (AMC) and skeletal muscle (SMC)-derived cell samples cultivated under 2D conditions and 3D spheroids.

For these samples we applied principal component analysis (PCA) (Figure 4). PCA was performed in the space of decimal log-transformed normalized gene expression levels (Figure 4A) and in the space of PALs (Figure 4B). The samples were primarily clustered according to both the type of tissue and the conditions of culturing, both at the gene and pathway levels.

### 2.3. Differential Gene Expression Analysis of AMC Myogenic Differentiation

#### 2.3.1. Analysis of Differentially Expressed Genes

To select the genes relevant in the context of myogenic differentiation in 2D/3D culturing conditions and AMCs/SMCs, we performed differential expression analysis in the following way. For each experimental group (2D and 3D AMC), we found differentially expressed genes (DEGs) in comparison between differentiated and non-differentiated cell cultures of that group. We used the DESeq2 package and a cutoff *p*-value of 0.1 and obtained different DEG sets for each comparison. The DEGs of each set are listed in Supplementary Table S1.

**Figure 5:**
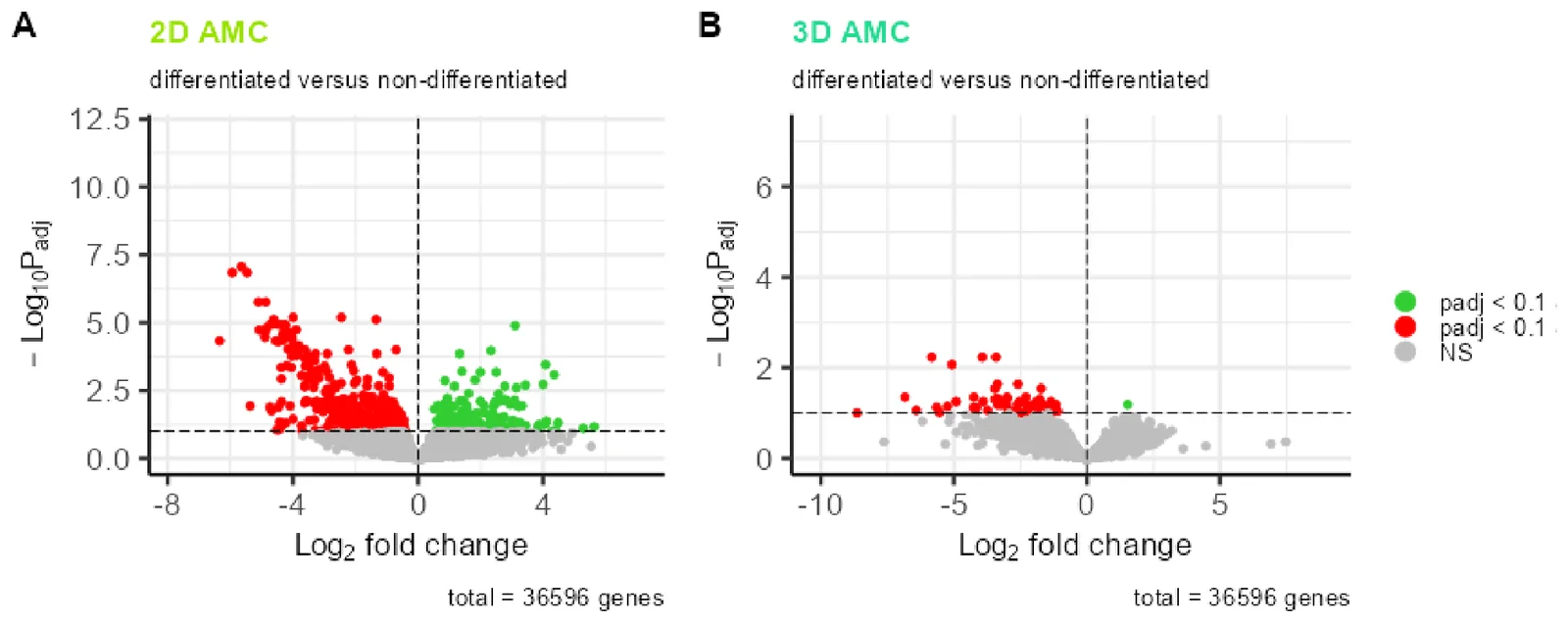
Differentially expressed genes (DEGs) in the 2D AMC (**A**) and 3D AMC (**B**) culturing conditions. The X-axis represents the fold change in gene expression in different samples and the Y-axis represents the −log10(adjusted *p*-value) as per the figure; red and green dots represent the DEGs that are significantly upregulated and downregulated in the differentiated cells, respectively. Not significantly differential genes are shown in grey.

To reveal the most significant DEGs related to AMC differentiation, we compared cells after myogenic differentiation and non-differentiated cells in 2D and 3D culturing conditions. In total, 703 statistically significant DEGs were identified for the two groups (differentiated vs non-differentiated) in 2D conditions. Among them, 256 genes were upregulated, and 447 genes were downregulated in the differentiated cells (Figure 5A). The top five up- and downregulated genes are listed in Table 2.

For 3D groups, we found 59 statistically significant DEGs, where only one gene, *PLEKHA6*, was upregulated and 58 genes were downregulated in the differentiated cells (Figure 5B). The top five upregulated and five downregulated genes for 2D conditions are listed Table 2 and one upregulated and five and downregulated genes for 3D a conditions are listed in Table 3.

#### 2.3.2. mRNA Validation of Myogenic Differentiation and RNA Sequencing Data

**Figure 6:**
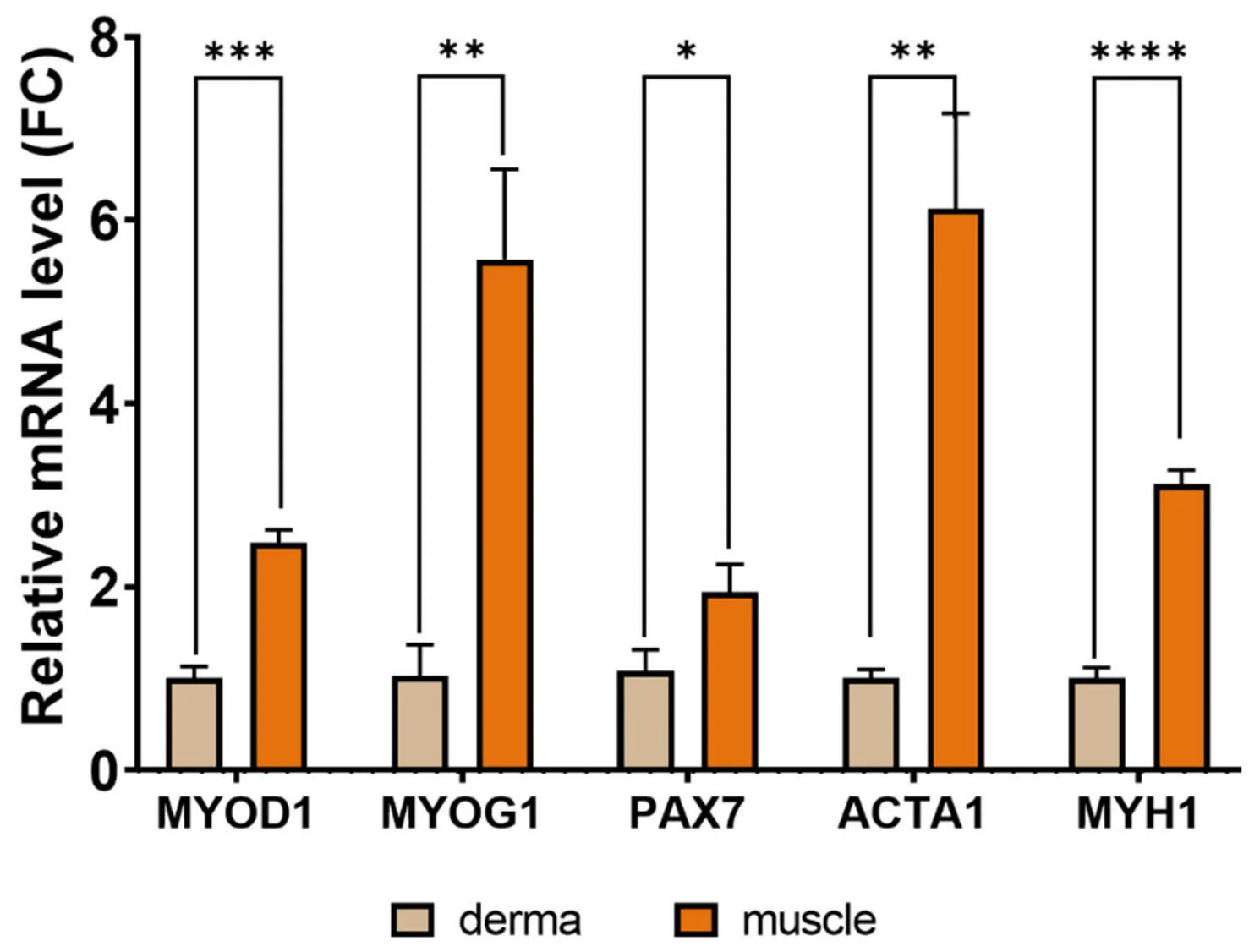
mRNA expression levels of *MYOD1*, *MYOG1*, *PAX7*, *ACTA1* and *MYH1* in dermal and muscle tissue. All statistical data are presented as the mean ± standard error of the mean (SEM). MYOD1 (***—*p* value = 0.000180); MYOG1 (**—*p* value = 0.001672); PAX7 (*—*p* value = 0.017898); ACTA1 (**—*p* value = 0.001060); MYH1 (****—*p* value = 0.000045) (Student’s *t*-test).

To confirm the validity of the detection of myogenic differentiation in primary tissue samples (non-cultivating cells) of the alveolar gingiva and skeletal muscle, an analysis of the expression of muscle cell markers (*MYOD1*, *MYOG1*, *PAX7*, *ACTA1* and *MYH1*) was performed. Increased expression of these genes was consistently observed only in samples of muscle tissue (Figure 6).

**Figure 7:**
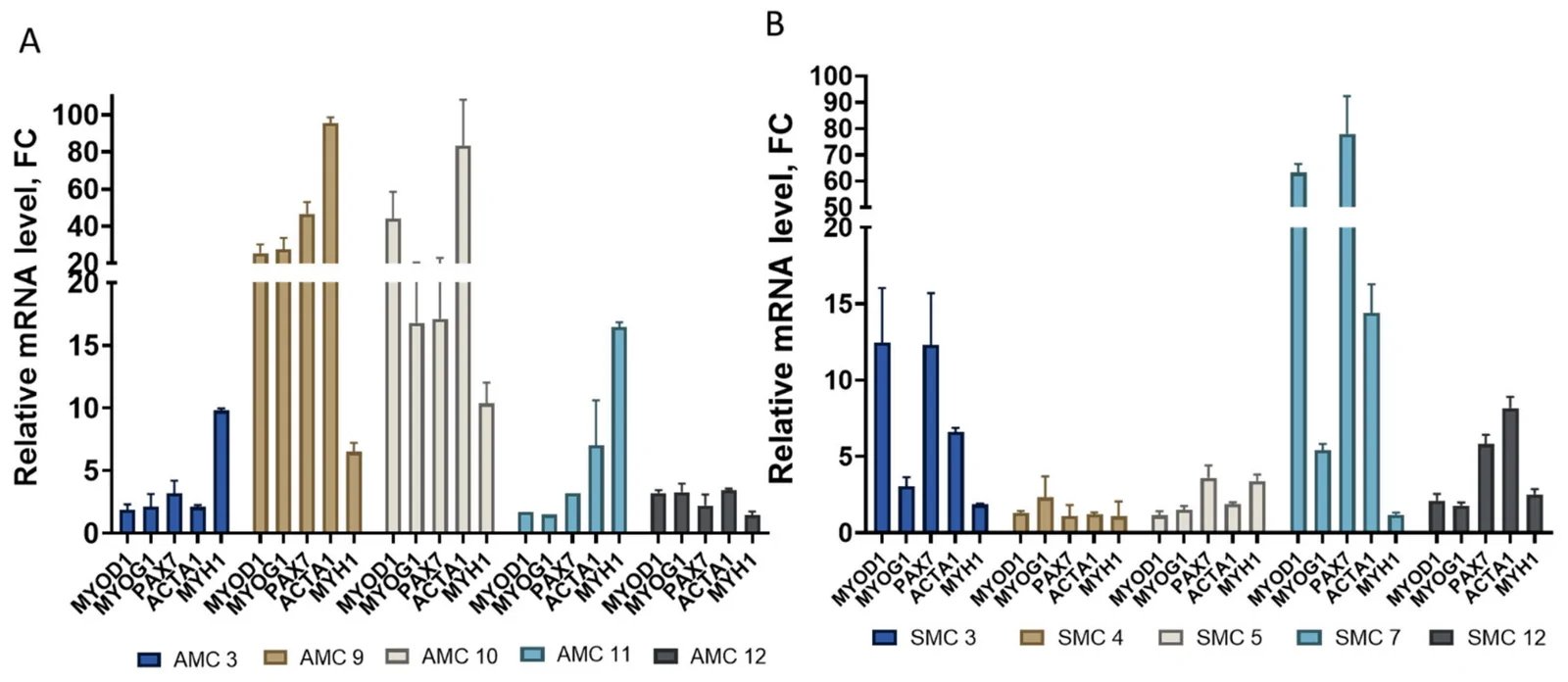
mRNA expression analysis of *MYOD1*, *MYOG1*, *PAX7*, *ACTA1* and *MYH1* in differentiated AMC (**A**) and SMC (**B**) cells in 2D conditions.

Next, we performed a comparative analysis of the mRNA expression of myogenic differentiation markers (*MYOD1*, *MYOG1*, *PAX7*, *ACTA1* and *MYH1*) in the initial samples and their 2D in vitro differentiated derivatives—both AMC (Figure 7A) and SMC (Figure 7B) cells.

**Figure 8:**
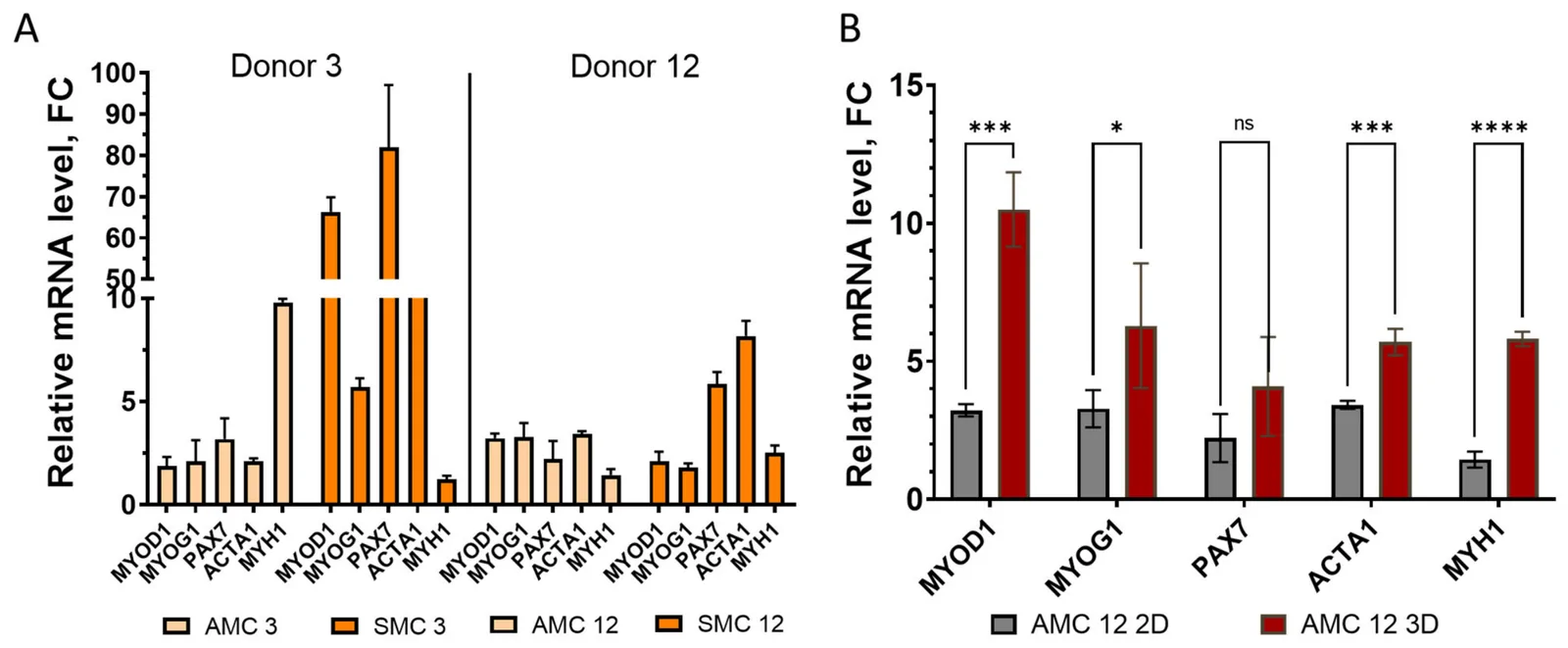
mRNA expression analysis of *MYOD1*, *MYOG1*, *PAX7*, *ACTA1* and *MYH1* in differentiated AMC and SMC cells from two donors cultured in 2D conditions (**A**) and differentiated AMC and SMC cells cultured in 2D and 3D (**B**). All statistical data are presented as the mean ± standard error of the mean (SEM). *MYOD1* (***—*p* value < 0.000001); *MYOG1* (*—*p* value = 0.002881); *PAX7* (*p* value = not significant, ns); *ACTA1* (***—*p* value = 0.018396); *MYH1* (****—*p* value = 0.000082) (Student’s *t*-test).

An increase in the expression of all myogenic markers was observed in both types of cells when differentiation was induced under 2D conditions, and their intensity depended on the specific sample/donor—whether it was cells of muscle origin (SMC) or alveolar gingiva (AMC). Therefore, we additionally conducted a comparative analysis of the expression of myogenic markers after 2D differentiation of cells obtained from the gingival and muscle tissues of the same donors (3, 12) (Figure 8A) and comparison of 2D and 3D differentiation conditions for one donor (12) (Figure 8B).

#### 2.3.3. Gene Ontology (GO) Annotation of DEGs

**Figure 9:**
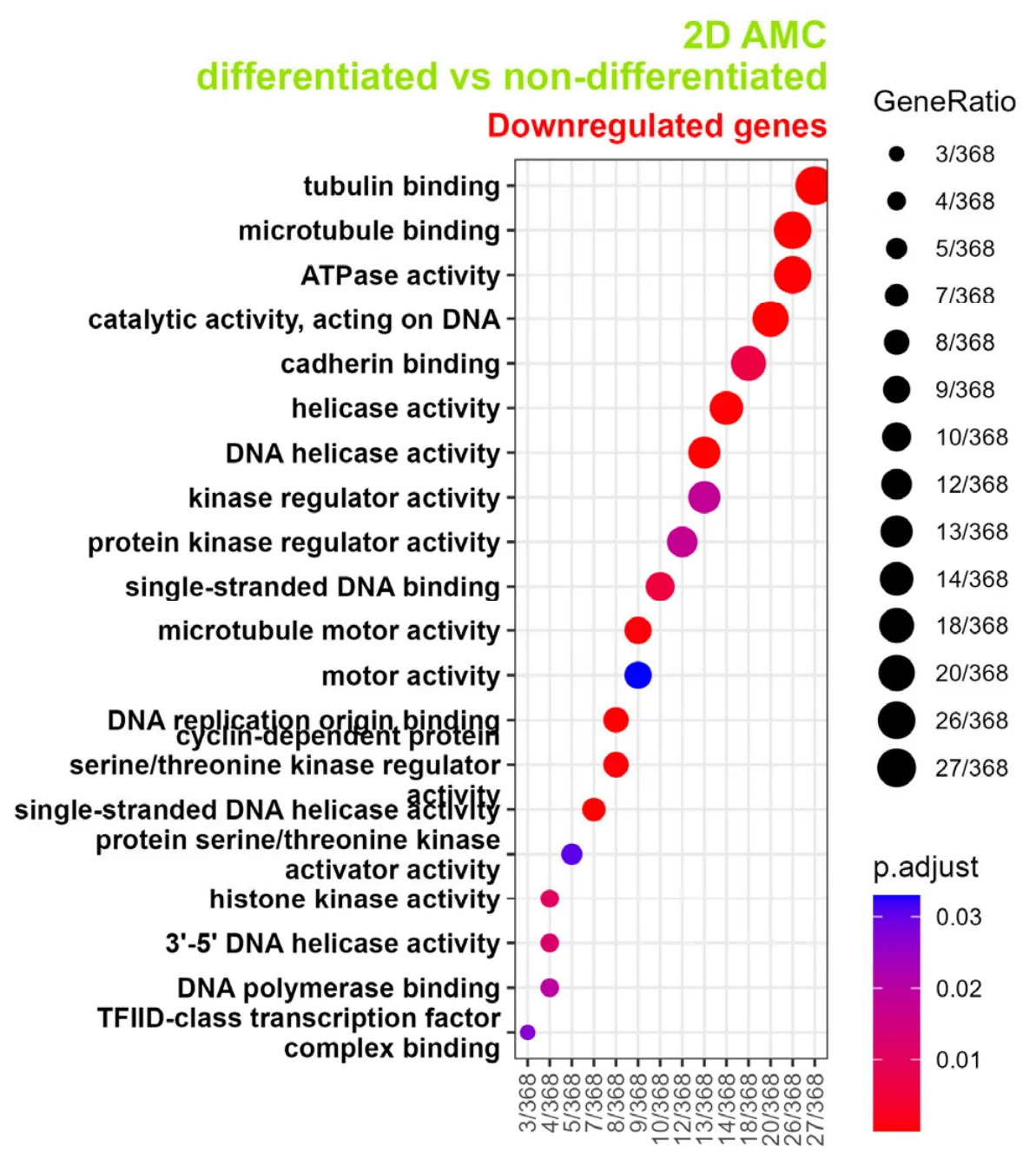
Gene Ontology terms enriched among the downregulated DEGs during myogenic differentiation in 2D AMC. The size of the circle represents the gene ratio, ratio between the number of DEGs in the observed experimental dataset (*p*-adj < 0.1, log2fc > 0 for upregulated genes or log2fc < 0 for downregulated genes) and the total number of genes in the respective GO term. Color represents the significance reflected by the q-value, with the default settings of the Benjamini–Hochberg false discovery rate.

Gene functions of DEGs in 2D conditions were annotated using GO enrichment analysis (Figure 9). DEGs downregulated in differentiated AMCs were significantly enriched in several biological processes, including tubulin binding, ATPase activity, cadherin binding, helicase activity, and motor activity GO terms (Figure 9). At the same time, no statistically significant GO terms were found among the upregulated genes. Similarly, no statistically significantly enriched GO terms could be found for the up- and downregulated differentiated AMC DEGs in 3D culturing conditions.

#### 2.3.4. Analysis of Molecular Pathways

**Figure 10:**
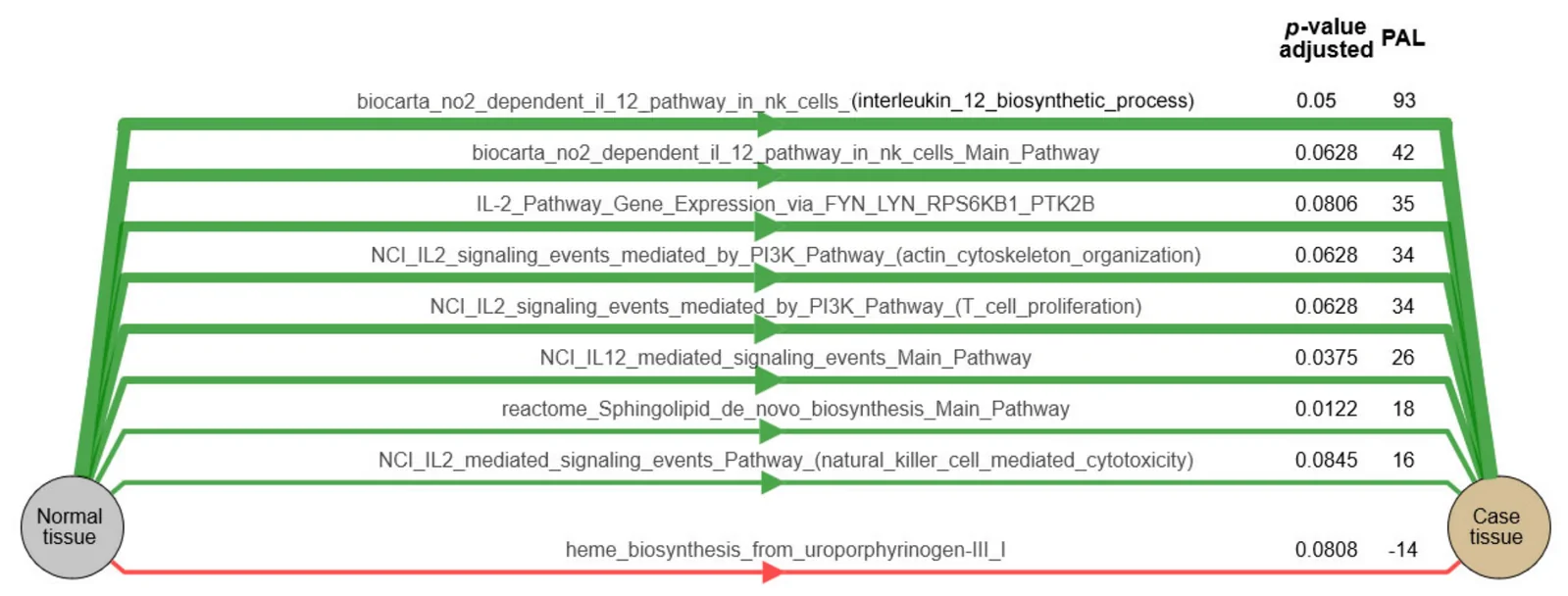
Activated (green) and inhibited (red) molecular pathways for the differentiated AMCs in 2D culturing conditions. Visualized with Oncobox pathway plotter software [13]. Indication “Normal tissue” means “non-differentiated cells”; “Case tissue” means “differentiated cells”.

In a comparison of differentiated and non-differentiated AMC cells in 2D culturing conditions, we found eight significantly upregulated molecular pathways and only one downregulated molecular pathway (Figure 10). The following pathways were activated in the differentiated cells: Biocarta NO2-dependent IL-12 pathway in NK cells—main pathway and its extension linked with the interleukin 12 biosynthetic process, NCI IL-12 mediated signaling events main pathway, three extensions of the NCI IL-2 mediated signaling events pathway: for natural killer cell mediated cytotoxicity, actin cytoskeleton organization, and T-cell proliferation, IL-2 pathway gene expression via *FYN LYN RPS6KB1 PTK2B*, and Reactome sphingolipid de novo biosynthesis main pathway. In turn, the pathway Heme biosynthesis from uroporphyrinogen-III I was inhibited in the differentiated cells.

**Figure 11:**
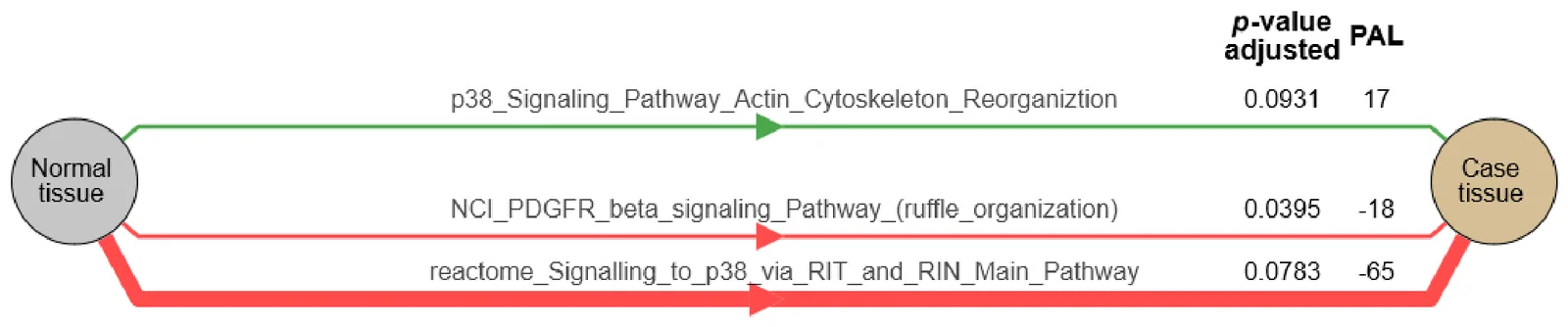
Activated (green) and inhibited (red) molecular pathways for the differentiated AMCs in 3D culturing conditions. Visualized with Oncobox pathway plotter software [13]. Indication “Normal tissue” means “non-differentiated cells”; “Case tissue” means “differentiated cells”.

For the comparison of differentiated and non-differentiated AMCs in 3D culturing conditions, we found only one upregulated and two downregulated molecular pathways (Figure 11): the P38 signaling pathway extension responsible for actin cytoskeleton reorganization was activated, and the pathways Reactome signalling to P38 via RIT and RIN and NCI PDGFR beta signaling pathway extension for ruffle organization were inhibited.

### 2.4. Comparison of AMC Differentiation in 2D and 3D Culturing Conditions

We then estimated the degree of similarity between differentially expressed gene sets obtained in the 2D and 3D culturing conditions. We separately intersected upregulated and downregulated genes and assessed the randomness of these intersections via a permutation test. For each intersected pair of experimentally observed DEGs, we randomly created 1000 pairs of gene sets of the same size. Then an intersection in each random pair of gene sets was performed and we calculated the empirical probability to obtain a greater number of common genes than in the experimentally observed pair. These empirical probabilities were denoted as *p*-values. That analysis was performed in the same way also for the up- and downregulated molecular pathways.

**Figure 12:**
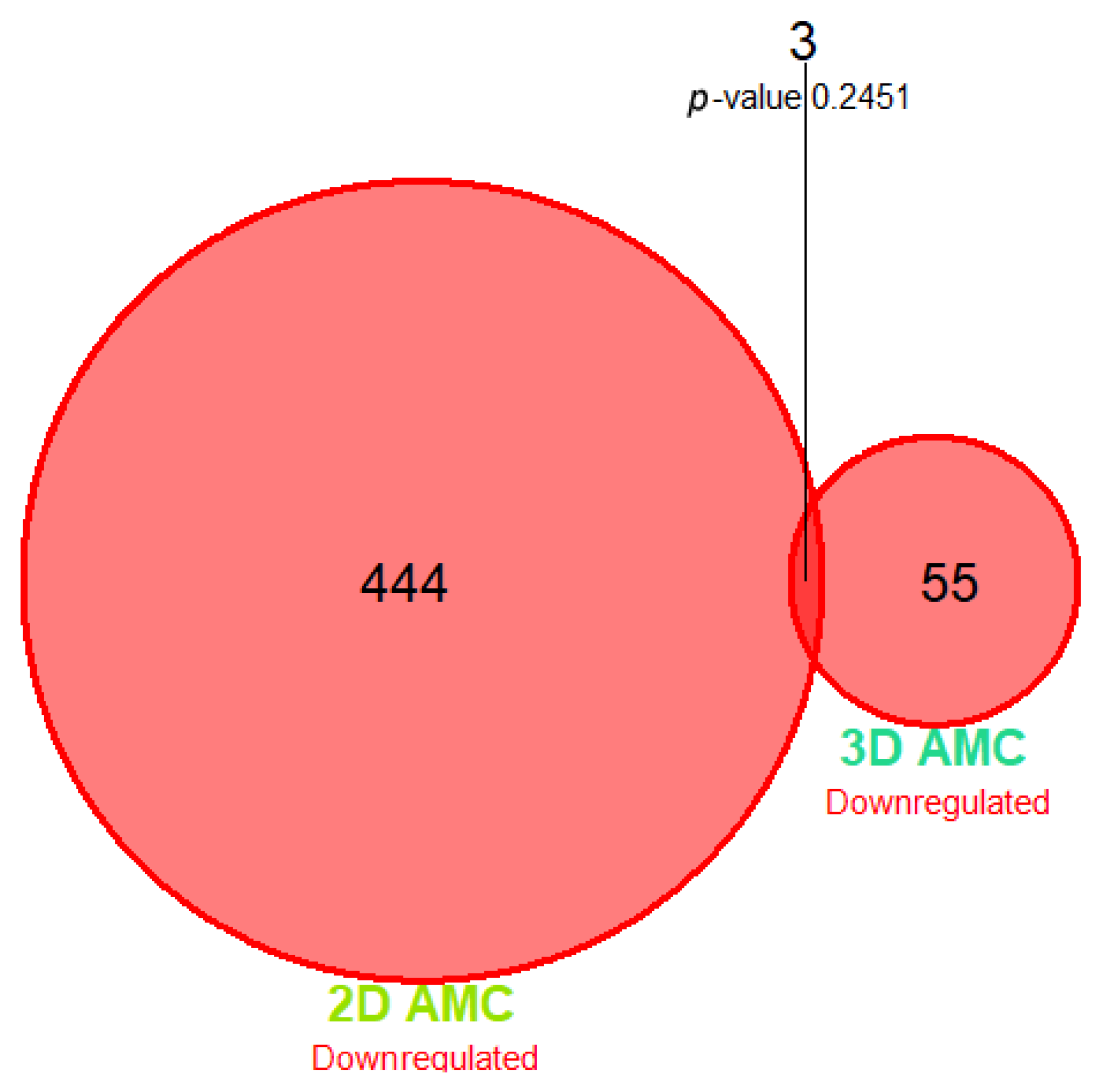
Downregulated genes in 2D and 3D culturing conditions of AMCs.

Then we assessed the similarity between DEGs during AMC differentiation in 2D and 3D conditions. To this end we separately intersected downregulated genes (447 in 2D and 58 in 3D) and upregulated genes (256 in 2D and 1 in 3D). We found only three common downregulated genes (*GAPDH*, *NPTX1*, *SPOCD1*) (Figure 12), which was not significant according to the permutation test described above. Taken together with the observation that there were no commonly activated pathways (Figure 10 and Figure 11), we conclude that the underlying molecular mechanisms of differentiation may be significantly different in the 2D and 3D AMC culturing conditions.

### 2.5. Comparison of AMC and SMC Differentiation

#### 2.5.1. 2D Culturing Condition

**Figure 13:**
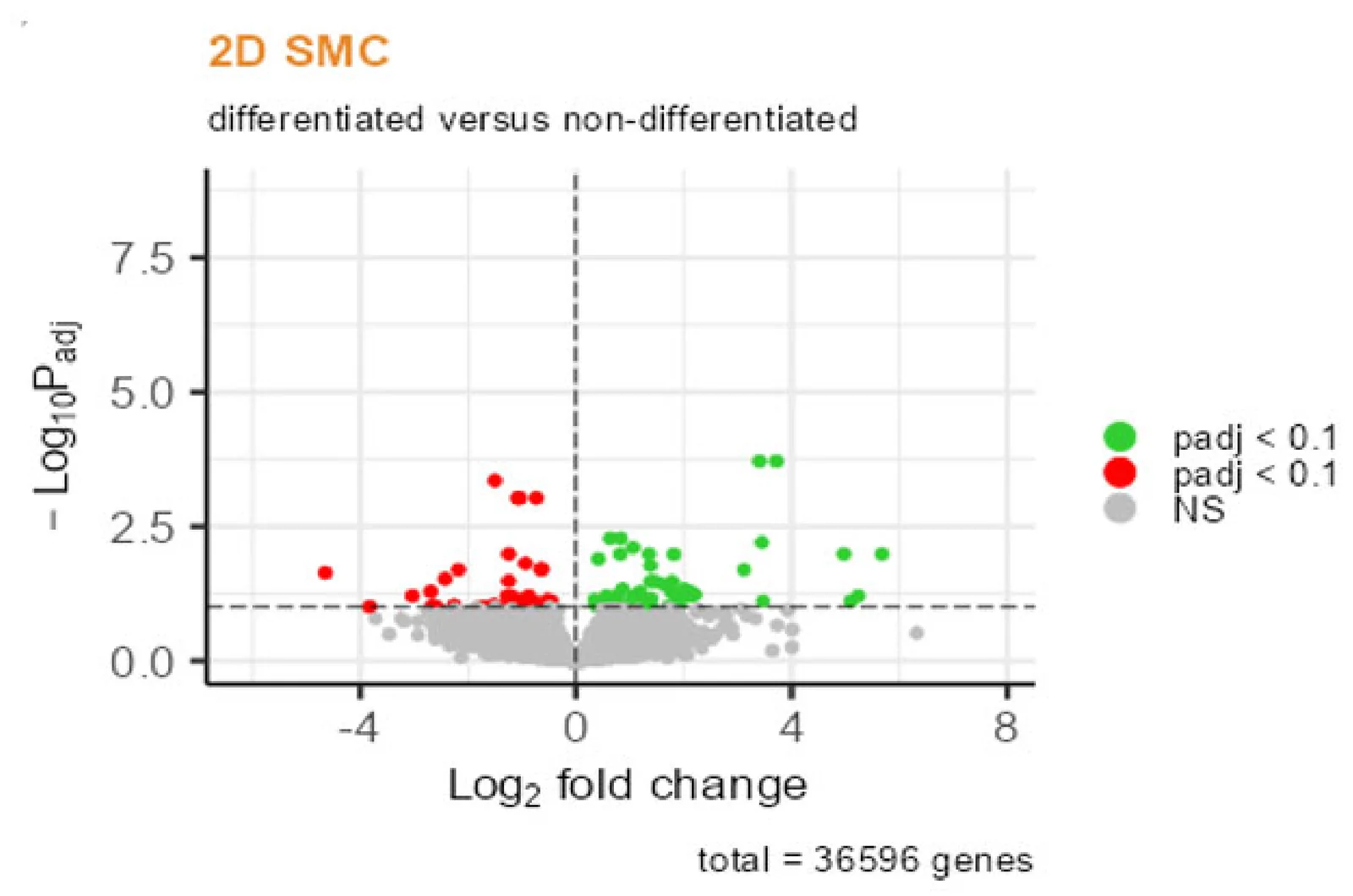
Differentially expressed genes (DEGs) in 2D SMC culturing conditions. The X-axis represents the fold change in gene expression in different samples and the Y-axis represents the −log10(adjusted *p*-value) as per the figure; red and green dots represent the DEGs that are significantly upregulated and downregulated in the differentiated cells, respectively. Not significantly differential genes are shown in grey.

We then compared differentiated and non-differentiated SMCs in 2D conditions. In total, 87 DEGs were detected, with 48 upregulated and 39 downregulated genes (Figure 13). The top five up- and downregulated genes are listed in Table 4.

**Figure 14:**
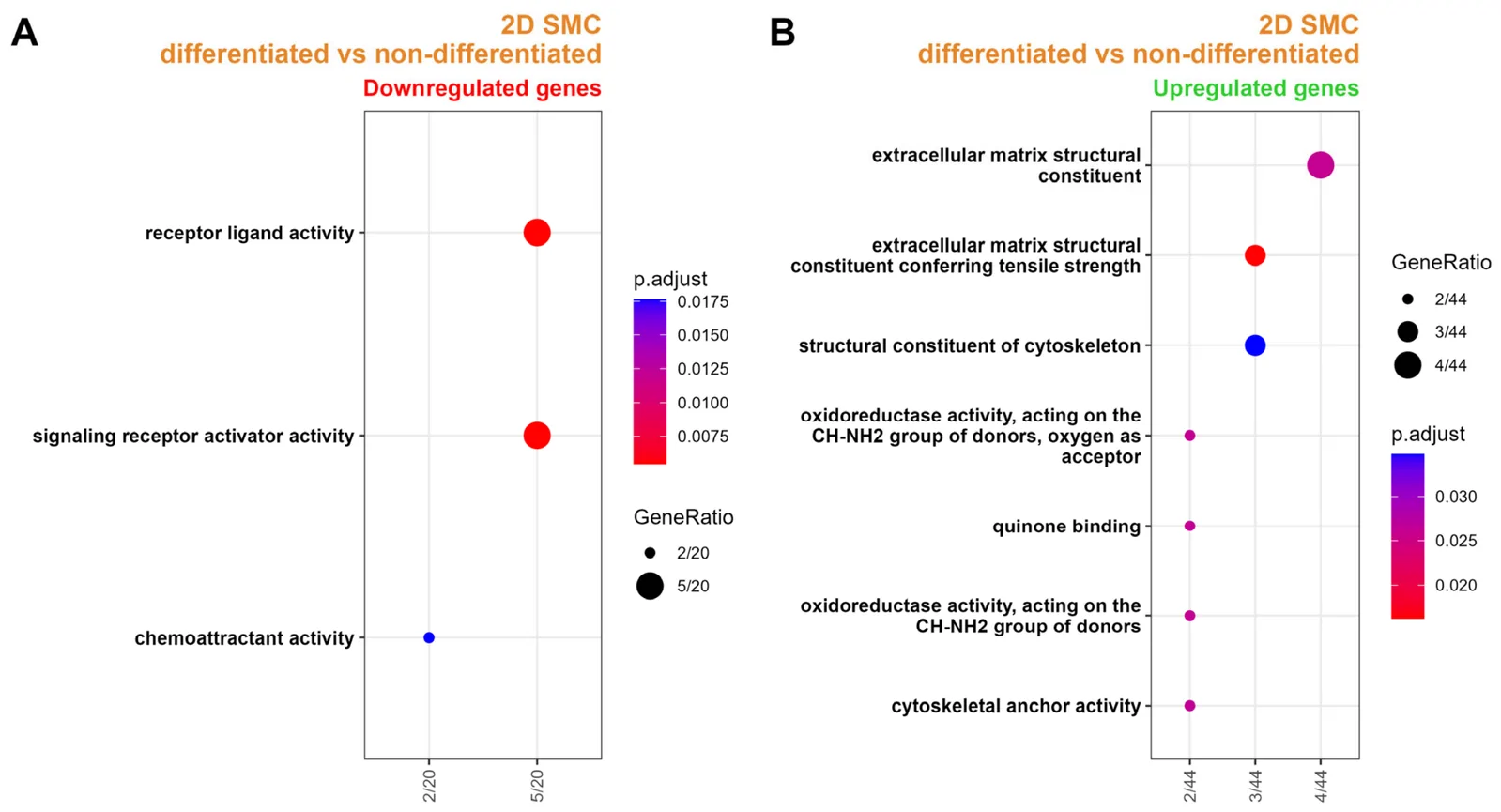
Gene Ontology terms enriched among the downregulated genes (**A**) and upregulated genes (**B**) during myogenic differentiation in 2D SMCs. The size of the circle represents the gene ratio. Color represents the significance reflected by the q-value, with the default settings of the Benjamini–Hochberg false discovery rate.

Gene functions of DEGs found in 2D conditions were also annotated using GO enrichment analysis (Figure 14). The downregulated DEGs were significantly enriched in the processes dealing with receptor ligand activity, signaling receptor activator activity, and chemoattractant activity (Figure 14A), whereas the upregulated genes were enriched in the extracellular matrix structural constituent, and oxidoreductase activity acting on the CH-NH2 group of donors processes (Figure 14B).

**Figure 15:**
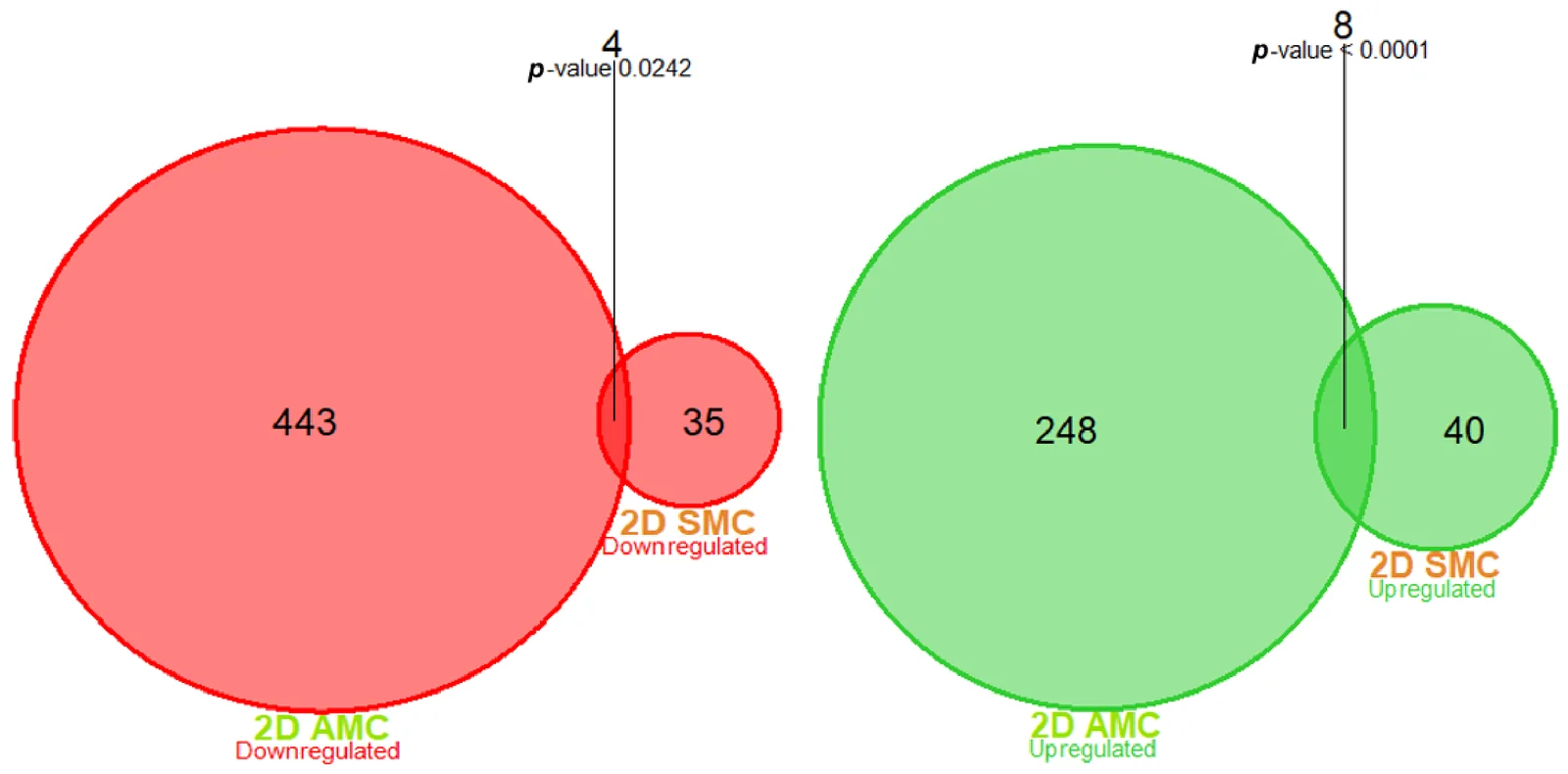
Downregulated genes (red) and upregulated genes (green) in 2D AMCs and SMCs.

We separately intersected downregulated genes (447 in AMCs and 39 in SMCs) and upregulated genes (256 in AMC and 48 in SMC). We found a total of four common downregulated (*CARD19*, *MIF*, *RN7SL518P*, *TCF7*) and eight common upregulated genes (*AOC3*, *ARRDC4*, *CACNA1H*, *CLSTN2*, *DAPK1*, *GPRC5B*, *HEATR5A*, *SLC40A1*), (Figure 15). The permutation test showed that this overlap was non-random in both cases (*p* < 0.05 in both cases), thus indicating a certain degree of similarity between AMC and SMC differentiation in 2D conditions. We could not perform an intersection test for differentially activated pathways because of the absence of differential pathways in 2D SMCs.

#### 2.5.2. 3D Culturing Condition

**Figure 16:**
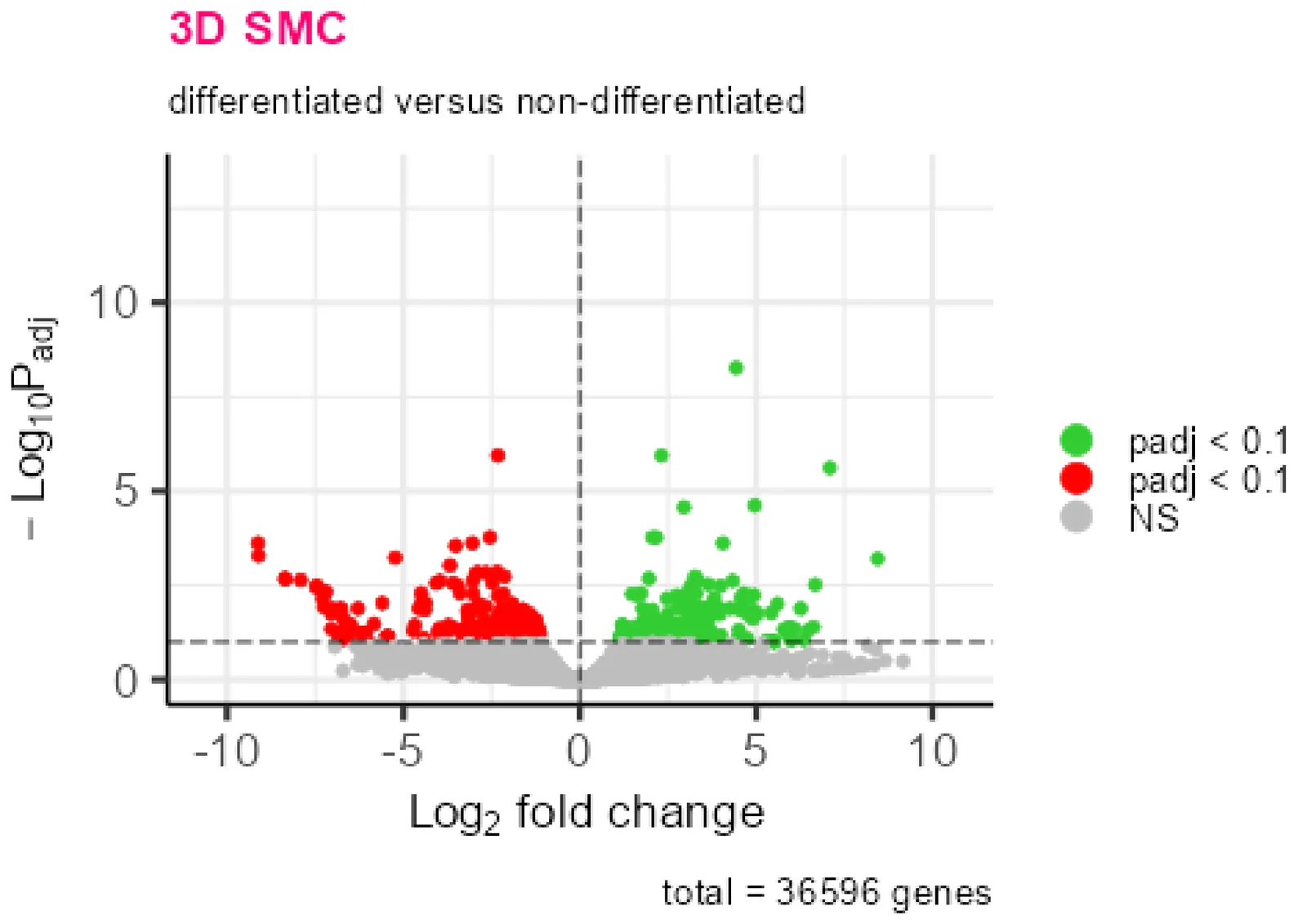
Differentially expressed genes (DEGs) in 3D SMC culturing conditions. The X-axis represents the fold change in gene expression in different samples and the Y-axis represents the −log10(adjusted *p*-value) as per the figure; red and green dots represent the DEGs that are significantly upregulated and downregulated in the differentiated cells, respectively. Not significantly differentially expressed are shown in grey.

**Figure 17:**
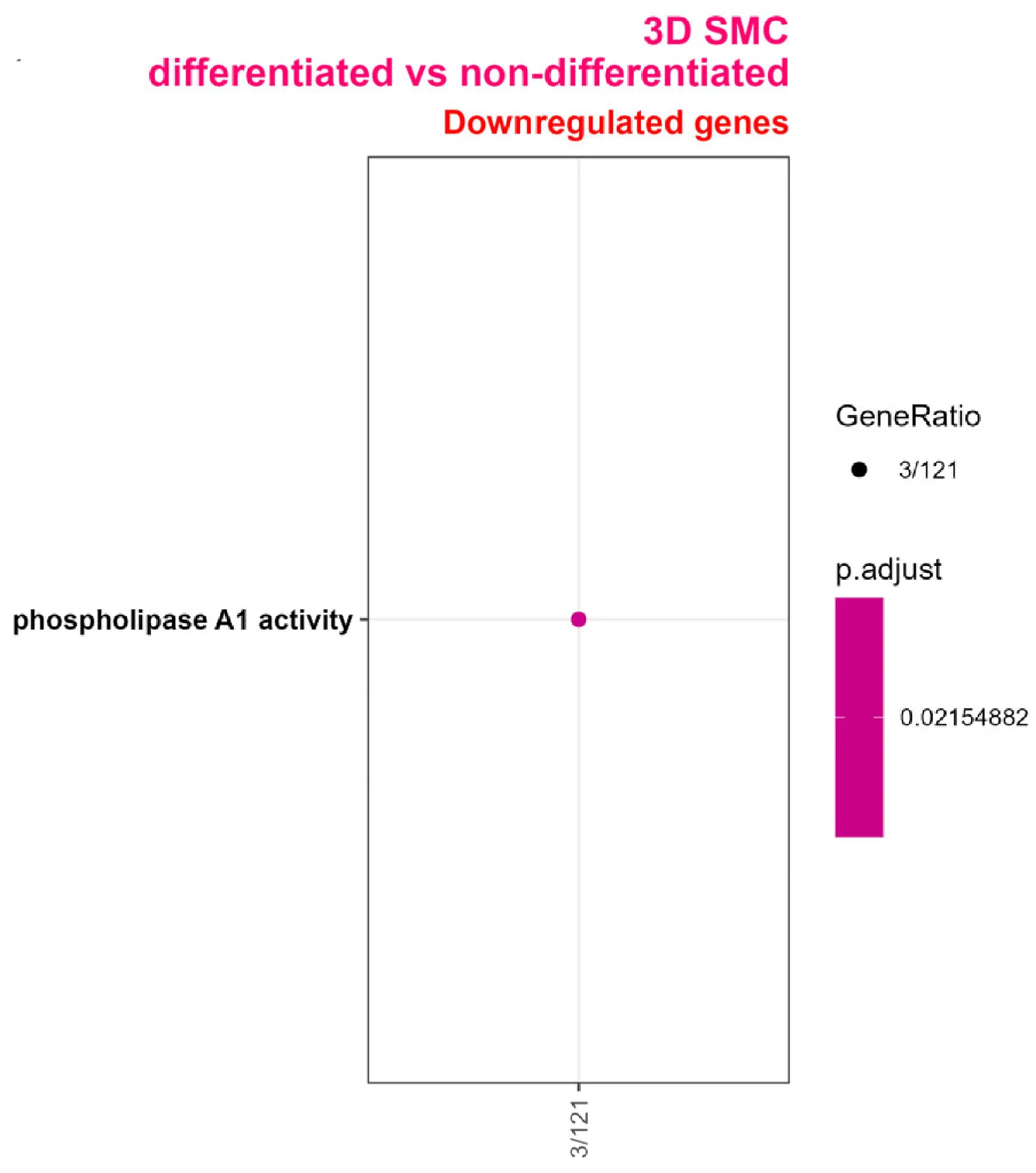
Gene Ontology term enriched among the downregulated genes during SMC myogenic differentiation in 3D culturing conditions. The size of the circle represents the gene ratio. Color represents the significance reflected by the q-value, with the default settings of the Benjamini–Hochberg false discovery rate.

In the same way we also analyzed SMC cells after myogenic differentiation in 3D culturing conditions. In total, 348 DEGs were detected between the differentiated and non-differentiated SMCs, where 185 genes were upregulated and 163 genes were downregulated (Figure 16). Top 5 up- and downregulated genes are listed in Table 5. Downregulated DEGs were significantly enriched in GO-term phospholipase A1 activity (Figure 17), whereas no GO terms were detected for the upregulated DEGs.

**Figure 18:**
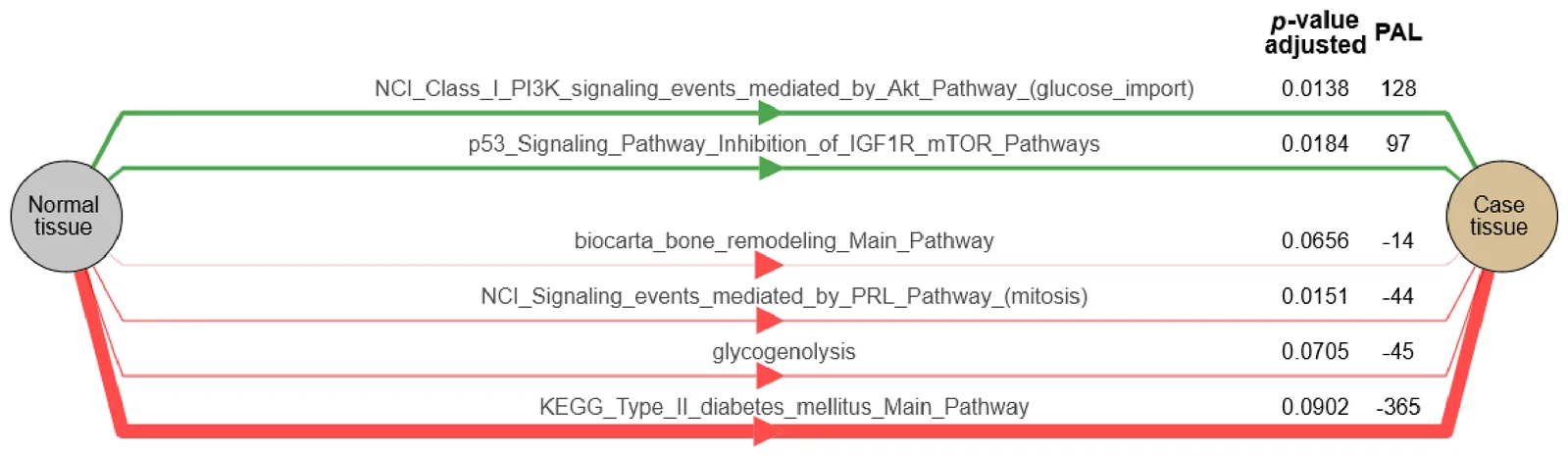
Activated (green) and inhibited (red) molecular pathways in the differentiated SMC samples in 3D culturing conditions.

At the level of pathway analysis, we detected 2 upregulated and 4 downregulated molecular pathways in differentiated SMCs in 3D culture conditions (Figure 18). Herein, the pathways NCI class I PI3K signaling events mediated by the Akt pathway (glucose import), and an extension of the P53 signaling pathway responsible for the inhibition of IGF1R mTOR pathways were activated, whereas the pathways for Biocarta bone remodeling, Glycogenolysis, KEGG type II diabetes mellitus, NCI signaling events mediated by PRL pathway (mitosis) were inhibited.

**Figure 19:**
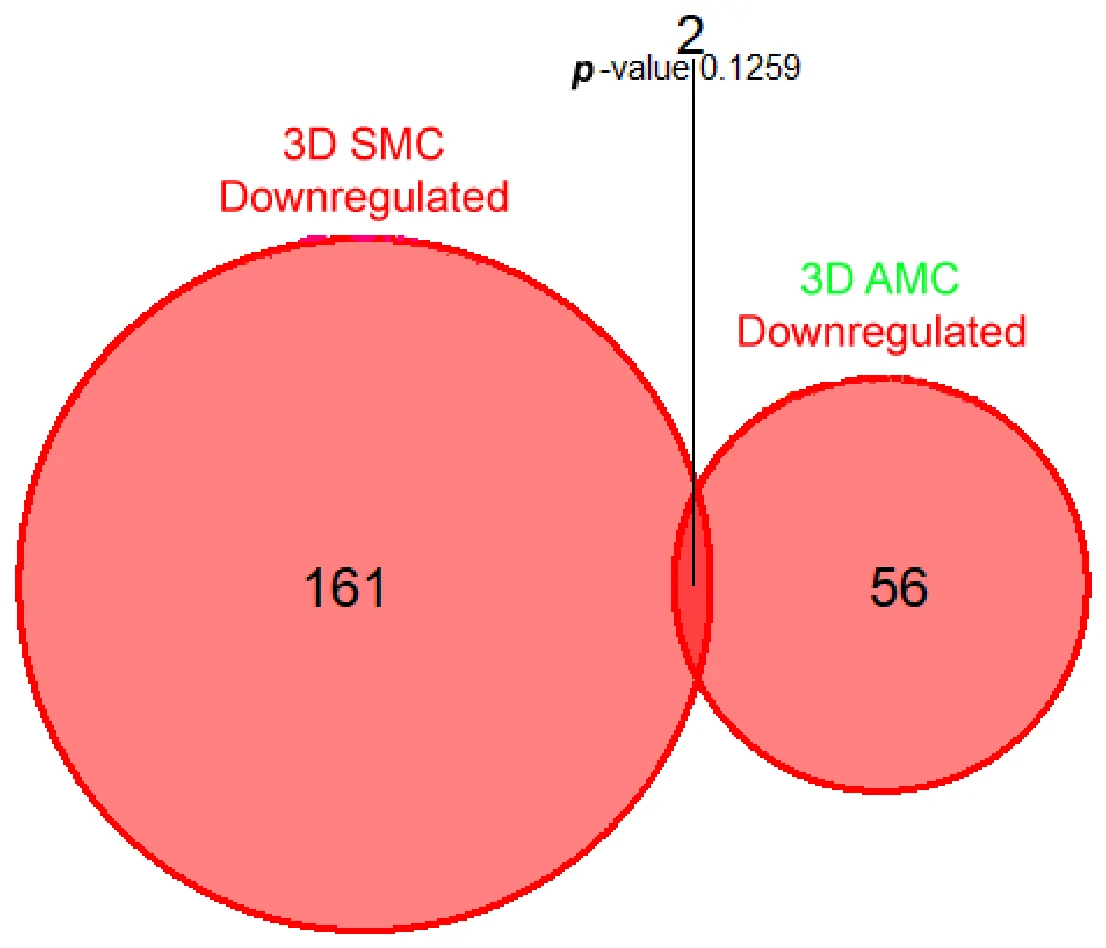
Downregulated genes in 3D AMCs and SMCs.

We then separately intersected downregulated genes (58 in AMC and 163 in SMC) and upregulated genes (1 in AMC and 185 in SMC). We found no common upregulated and only two common downregulated genes: *CTHRC1* and *HPD* (Figure 19), which were assessed as random events by the permutation test.

**Figure 20:**
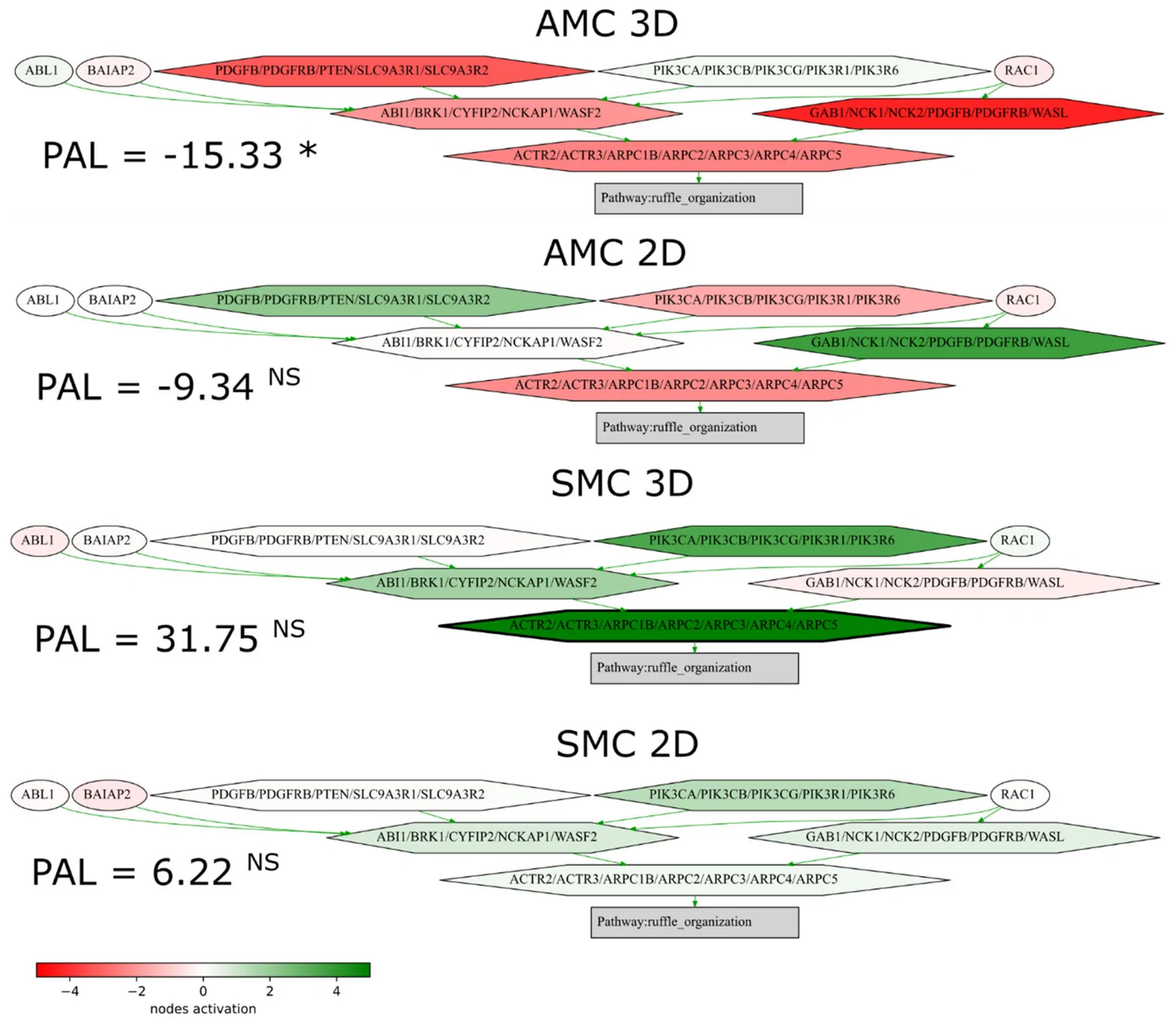
PDGFR beta signaling pathway (ruffle organization) shown as an interacting network separately in AMCs and SMCs. Green/red arrows indicate activation/inhibition interactions, respectively. The color depth reflects the extent of node activation (natural logarithms of the expression fold change for each node; the reference is the geometric average between expression levels in all samples in the respective groups). PAL—pathway activation level; *—adjusted *p*-value < 0.05; NS—non-significant.

Finally, we compared the differentiation-associated changes in PDGFRβ signaling across AMCs and SMCs cultured under 2D and 3D conditions. The NCI PDGFR beta signaling pathway extension responsible for ruffle organization was significantly inhibited in differentiated 3D AMCs, whereas its changes did not reach statistical significance in 2D AMCs or in 2D and 3D SMCs (Figure 20).

## 3. Discussion

Myoblast migration, adhesion, and fusion play a critical role in the development and regeneration of muscle tissue upon injury. In this study, we used AMC cells as a source of myogenic cells due to their high proliferative capacity and stability. The process of myogenesis is regulated by several specific signaling pathways and is mediated by different intracellular and cell-surface proteins [14]. However, the exact mechanisms guiding progenitor cells to myogenesis remain uncertain and controversial. In this report, we attempted to shed light on molecular mechanisms governing the differentiation of AMCs to multinucleated myotubes.

In this study we found that molecular processes encompassing extracellular matrix (ECM) structural constituents and oxidoreductase remodeling are interconnected with myogenic differentiation of human alveolar mucosa-derived cells. In the previous literature, the following relation was reported.

ECM structural constituents are closely associated with myogenic, and particularly myofibroblast, differentiation of lung-derived cells. ECM composition and stiffness regulate cell differentiation through integrin/FAK-dependent mechanotransduction and TGF-β signaling, promoting cytoskeletal remodeling, expression of α-SMA and acquisition of a contractile phenotype. Importantly, alveolar epithelial cells can undergo mesenchymal transition in an ECM-dependent manner, while fibronectin/integrin signaling facilitates myofibroblast differentiation [15,16]. Thus, enrichment of ECM structural constituent genes may reflect establishment of a matrix-producing, contractile phenotype characteristic of myogenic/myofibroblast differentiation.

In turn, oxidoreductase activity may contribute to this process through ROS-dependent signaling and ECM maturation. TGF-β strongly induces NOX4 in human lung mesenchymal cells and fibroblasts, and NOX4-derived ROS are required for induction of α-SMA, collagen and other features of myofibroblast differentiation [17,18]. Mitochondrial ROS additionally contribute to TGF-β-induced profibrotic signaling [19]. Oxidoreductases of the LOX/LOXL family provide a direct connection between these two functional categories: they cross-link ECM proteins and increase matrix stiffness, while LOXL2/LOXL3 enzymatic activity is required for lung fibroblast-to-myofibroblast transition [20]. Therefore, simultaneous enrichment of ECM structural constituents and oxidoreductase activity is consistent with activation of an interconnected TGF-β–ROS–ECM–mechanotransduction program accompanying myogenic/myofibroblast differentiation.

### 3.1. AMC Differentiation

The present analysis revealed several potential molecular players involved in AMC differentiation in 2D and 3D culturing conditions.

#### 3.1.1. 2D Culturing Condition

In 2D conditions five top upregulated genes in differentiated cells were *UNC5D*, *LRRC7*, *H19*, *LINC01121*, and *RGS5*, and five most strongly downregulated genes were *HIST1H3G*, *CDC20*, *HIST1H2AJ*, *HIST1H3B*, and *NEK2.* The UNC-5 family of netrin receptors is known to regulate axon guidance and cell survival [21], as well as cell-cell adhesion, guidance, and migration [22]. It is a candidate gene for neurodevelopment [23] and for promoting inhibition of cell metastasis by activating death-associated protein kinase 1 in prostate cancer [24]. Genes of this family have tumor-suppressive function and are frequently downregulated in renal cancers due to promoter hypermethylation and loss of heterozygosity [25]. *UNC5D* was previously described as the factor essential for osteogenic differentiation of human adipose-derived stem cells [26].

In turn, overexpression of long noncoding RNA *H19* regulates osteogenic differentiation of bone marrow stem cells via targeting miRNA-140-5p/Wnt1 signaling pathway [27] and via miR-532-3p/SIRT1 axis [28]. Also, it regulates the adipogenic differentiation of bone mesenchymal stem cells (BMSCs) [29].

Among the top downregulated genes, *CDC20* and *NEK2* stand out. Cell division cycle 20 homolog (Cdc20) is an essential cell-cycle regulator required for the completion of mitosis [30] 202 whereas Nek2 is a mitotic Ser/Thr kinase that localizes predominantly to centrosomes and kinetochores and orchestrates centrosome disjunction and faithful chromosomal segregation. Its activity is tightly regulated during the cell cycle with the help of several other kinases and phosphatases and via proteasomal degradation [31]. In line with this, the downregulated genes were enriched for GO terms microtubule binding, cyclin-dependent protein serine/threonine kinase activator activity, and 3′-5′ DNA helicase activity. For the other top differentially expressed genes, we found no published associations between *LINC01121*, *LRRC7*, *RGS5*, *HIST1H3G*, *HIST1H2AJ*, *HIST1H3B* and cell differentiation.

We propose that the observed expression pattern is consistent with a transition from a proliferative progenitor state toward a differentiated, regeneration-associated phenotype, although the strength of evidence differs markedly among these genes. The strongest direct link is H19, a myogenesis-associated lncRNA induced during human and mouse satellite-cell differentiation and muscle regeneration; it promotes differentiation through miR-675-3p/5p-mediated repression of the anti-myogenic gene SMAD1/5 and the DNA-replication factor CDC6 [32], and also regulates the satellite-cell/imprinted-gene network during muscle regeneration [33]. In parallel, downregulation of CDC20 and the mitotic kinase NEK2, together with replication-dependent histones HIST1H3G, HIST1H3B, and HIST1H2AJ, is compatible with cell cycle withdrawal and reduced DNA replication/chromatin assembly, a prerequisite for terminal myogenic differentiation; notably, H19 itself couples myogenic differentiation to suppression of DNA-replication machinery. By contrast, UNC5D, LRRC7, LINC01121, and RGS5 should not currently be described as established muscle-regeneration genes: their upregulation is more reasonably interpreted as accompanying changes in cell identity, signaling and cytoskeletal/mesenchymal organization, with RGS5 particularly associated with vascular smooth-muscle/pericyte-like states. Thus, the combined signature—especially H19 induction plus coordinated repression of mitotic and replication-associated genes—supports differentiation and acquisition of a regeneration-related phenotype, whereas the contribution of UNC5D, LRRC7, LINC01121 and RGS5 remains suggestive rather than mechanistically established.

Most of the upregulated molecular pathways in 2D AMC were related to IL-12 and IL-2 signaling events. IL-12 is primarily associated with the differentiation of Th1 cells, while IL-2 is important for the proliferation and differentiation of various T cell subsets [34]. However, one downregulated pathway was connected with heme biosynthesis.

#### 3.1.2. 3D Culturing Condition

The five most strongly downregulated genes in differentiated 3D AMC cells were *MT3*, *TUBB2B*, *DLK1*, *ISM2*, and *RASGEF1A*, whereas *PLEKHA6* was the only upregulated gene. Among them, the *MT3* gene is known to be specifically upregulated in the differentiation process of C2C12 myoblasts treated with bone morphogenetic protein 4 (BMP4) and *MT3* knockdown inhibited BMP4-induced osteoblast differentiation in C2C12 cells [35]. There is limited evidence linking *TUBB2B*, *DLK1*, *ISM2*, *RASGEF1A*, and *PLEKHA6* to cell differentiation. Thus, their role in differentiation may be specific to AMC in 3D culture.

The differential regulation of PDGFRβ signaling across the experimental conditions suggests that its downregulation may represent a specific feature of AMC differentiation, particularly in 3D culture. Given the established role of PDGFR signaling in mesenchymal cell differentiation [36,37], this finding further emphasizes the dependence of the AMC differentiation program on cell origin and culture context.

### 3.2. Comparison of Culturing Conditions for AMC Differentiation

Another goal of our study was to investigate whether the differentiation in 2D and 3D culturing conditions is similar in AMCs and SMCs. We observed a significant difference in AMC differentiation patterns in different cultivation conditions. The number of common differential genes and pathways was small, and a random permutation test showed that the overlap of differential genes and pathways between the two datasets was not significant. This observation highlights the importance of the culturing conditions for this type of experiment. In line with our results, it was shown previously that 3D cell culturing techniques may be dramatically different than 2D methods, and also strongly beneficial compared to 2D culturing conditions [38].

### 3.3. Similarities Between AMC and SMC Differentiation

We also investigated similarities in gene expression changes during AMC and SMC differentiation. To do this, we intersected differential genes in AMCs and SMCs for 2D and 3D conditions.

#### 3.3.1. 2D Culturing Condition

In 2D culturing conditions, AMC and SMC differentiation showed a significant degree of similarity. We identified four shared downregulated genes (*CARD19*, *MIF*, *RN7SL518P*, and *TCF7*) and eight shared upregulated genes (*AOC3*, *ARRDC4*, *CACNA1H*, *CLSTN2*, *DAPK1*, *GPRC5B*, *HEATR5A*, and *SLC40A1*), with both overlaps being statistically significant. TCF7 is a transcription factor involved in the Wnt signaling pathway [39], which plays an important role in cell differentiation [40]. *CARD19* and *MIF* are primarily associated with immune and inflammatory responses rather than being direct regulators of differentiation [41]. For the other shared genes, direct involvement in myogenic differentiation has not been established. Nevertheless, the statistically non-random overlap, together with the induction of established myogenic markers in both cell types, suggests that AMC and SMC partially converge toward a common transcriptional state during myogenic differentiation. At the same time, the relatively small number of shared DEGs indicates that AMCs do not simply reproduce the SMC differentiation program but retain a substantial cell-type-specific component. Thus, our findings support the current concept of muscle regeneration as a coordinated but context-dependent process involving both common myogenic mechanisms and cell-origin-specific molecular responses.

#### 3.3.2. 3D Culturing Condition

In 3D culturing conditions, differentiation of AMCs and SMCs was largely different and showed only two common downregulated genes (*CTHRC1* and *HPD*), whereas no common upregulated genes were identified. Moreover, the overlap of downregulated genes was not statistically significant according to the permutation test. *CTHRC1* is associated with cell migration, invasion and wound healing in stomach tissues [42] and may affect immune regulation, extracellular matrix deposition, and angiogenesis during wound healing [43]. For *HPD*, we found no evidence of its association with cell differentiation. Thus, in contrast to the significant convergence of AMC and SMC transcriptional responses observed in 2D conditions, 3D culturing appears to enhance cell-type-specific differentiation responses. This may reflect differences between AMCs and SMCs in their responses to the three-dimensional microenvironment, including cell–cell and cell–matrix interactions and spatial organization. Importantly, these findings do not necessarily indicate impaired myogenic differentiation in 3D, but rather suggest that its underlying molecular mechanisms become more dependent on cell origin and culture context.

Taken together, our transcriptomic data suggest that AMC myogenic differentiation should not be viewed as activation of a single canonical molecular program. Rather, acquisition of the myogenic phenotype is accompanied by coordinated cell cycle withdrawal, remodeling of extracellular matrix and signaling processes, and changes in differentiation-associated genes. The significant transcriptional overlap between AMCs and SMCs in 2D indicates partial convergence toward a common myogenic state, whereas the substantially different responses observed in 3D demonstrate that this process remains strongly dependent on cell origin and culture context. Thus, AMCs appear capable of acquiring a myogenic phenotype without simply reproducing the molecular differentiation program of muscle-derived stromal cells, which may be important when considering AMCs as an alternative cell source for muscle regenerative applications.

## 4. Materials and Methods

### 4.1. Ethical Consideration

The study was approved by the Local Ethical Committee of Pirogov National Medical and Surgical Center, Moscow, Russia (Protocol#2 from 17 February 2022) following the WMA Declaration on Helsinki of Ethical Principles for medical research involving human subjects and Good Clinical Practice Guidelines approved by the Russian Ministry of Health.

### 4.2. Experimental Biosamples

Biopsies (2–3 mm^3^) of the alveolar (flexible) mucosa from the retromolar area and attached (fixed) gingiva from the distal mandibular molar level were performed under local anesthesia (Sol. Articaini 40 mg/mL, 1.7 mL). All biopsy samples were placed in a sterile medium containing α-MEM (Sigma, St. Louis, MO, USA), 2% FBS (HyClone, Logan, UT, USA), penicillin (200 U/mL), streptomycin (200 mg/mL), and amphotericin (200 U/mL) (Stem Cell Technologies, Vancouver, BC, Canada), labeled and delivered at +4 °C to the laboratory within 2 h.

### 4.3. Isolation and Cultivation of Cells

All biopsy samples were processed using a similar protocol as follows. AMCs were isolated from alveolar mucosa, AGCs from attached gingiva, and skeletal muscle cells (SMCs) from skeletal muscle tissue. The biopsy samples were washed with α-MEM (Sigma) supplemented with gentamicin (50 µg/mL) (Sigma). A sample was transferred into a 15-milliliter tube in α-MEM supplemented with 10% FBS (HyClone), gentamicin (20 µg/mL), 0.05% solution of collagenase type II (Sigma) and incubated for 12 h at 37 °C. Before enzymatic digestion, skeletal muscle samples were minced. After that cell suspension was centrifuged at 200× *g* for 10 min. The cell pellet was resuspended in culture medium DMEM/F12 (HyClone) supplemented with 20% FBS (HyClone), gentamicin (20 µg/mL) and plated in culture flasks (Nunc A/S, Denmark) at a density of 3–5 × 10^4^ cells/cm^2^. Cells were cultured at 37 °C, 95% humidity and 5% CO_2_ with the medium changed every 3 days. Cell cultures were passaged at 50% confluence. Visual observation of culture growth and cell morphology was conducted by phase contrast microscopy. Cell cultures at passage 2 were used for research experiments.

### 4.4. Myogenic Differentiation

To induce myogenic differentiation, AMC, AGC and SMC were seeded at passage 2 at a density of 5 × 10^5^ cells/cm^2^ and cultured in DMEM/F12 supplemented with 20% FBS (HyClone) until a 90–100% confluent monolayer was formed. Then culture medium was replaced with low-glucose DMEM (HyClone) supplemented with 2% horse serum (BioInd, Beit Haemek, Israel) and cells were incubated for 5–10 days at 37 °C, 95% humidity and 5% CO_2_ until myotube-like structures formed.

### 4.5. 3D Culture

To prepare cell suspensions, monolayer cultures at passage 2 were enzymatically treated with a mixture of 0.25% trypsin and EDTA (0.2 g/L in PBS) solutions (1:1) for 1 min. After that cell suspension was centrifuged at 100× *g* for 7 min. The cell pellet was resuspended in culture medium (DMEM/F12 supplemented with 20% FBS) to reach a concentration of 3.3 × 10^6^ cells/mL, and placed into non-adhesive agarose plates prepared using a 3D Petri Dish (Microtissue, Providence, RI, USA). The filled agarose plates were transferred into 12-well plates (SPL, Pocheon-si, Republic of Korea). Spheroids were cultured under standard conditions for 5–7 days at 37 °C, 95% humidity and 5% CO_2_ with medium changes every 2–3 days. To induce myogenic differentiation, the culture medium was replaced with low-glucose DMEM (HyClone) supplemented with 2% horse serum (BioInd) and spheroids were incubated for 7 days at 37 °C, 95% humidity and 5% CO_2_.

### 4.6. Immunofluorescence Staining and Western Blot Analysis

For fluorescent analysis of actin, MyoD1, and myogenin cells were fixed with 4% PFA. Primary monoclonal anti-human mouse antibodies for muscle actin (M0635, clone HHF35, DAKO, Carpinteria, CA, USA), MyoD1 (M3512, clone 5.8A, DAKO), myogenin (M3559, clone F5D) and secondary goat anti-mouse IgG (H + L) highly cross-adsorbed Alexa Fluor™ 546-labeled (A-11030, Invitrogen, Carlsbad, CA, USA) antibody were used. Nuclei were stained with DAPI (32670, Sigma-Aldrich). Images were acquired using a fluorescent Axioplan 2 microscope with an Axiocam HRc camera and AxioVision software V 4.6.3.0 (Carl Zeiss, Oberkochen, Germany). Whole-cell extracts were prepared in ice-cold Lysis-M Reagent (4719964001, Roche, Basel, Switzerland) with protease inhibitors (04693116001, Roche). Protein concentrations were determined using a protein assay system (5000205, Bio-Rad, Hercules, CA, USA). Immobilon-FL PVDF membrane (IPFL00010, Merck, Darmstadt, Germany) for protein blotting was used. Membranes were incubated with the same primary antibodies as in the immunofluorescence assay. Pan-actin antibody (MABT1333, clone 2A3, Merk) was used for loading control. Goat anti-mouse IgG (H + L) highly cross-adsorbed Alexa Fluor™ 488-labeled secondary antibody (A-11029, Invitrogen) was used for visualization. Band detection was performed using a variable mode imager Typhoon9410 (GE Healthcare, Chicago, IL, USA). Quantitation of protein bands was carried out using TotalLab v.2.01 software (Newcastle upon Tyne, UK).

### 4.7. Estimation of the Efficiency of Myogenic Differentiation

To estimate the efficiency of myogenic differentiation, cells seeded on slide glasses were fixed with ice-cold methanol. The cells were simultaneously incubated with the primary murine antibodies specific to sk-actin (M0635, clone HHF35, DAKO). Then the cells were incubated with the secondary Alexa Fluor™ 546-labeled rabbit antibodies against murine antibodies (Life Technologies, Carlsbad, CA, USA). Images were visualized and photographed by means of an Axioplan 2 microscope with an Axiocam HRc camera, and AxioVision software (Carl Zeiss, Germany). Alexa Fluor™ 546-stained areas were separated, red-filled, converted to black-and-white BMP format and a ratio between the specifically stained area and the total field area was estimated on photographs using ImageJ software 1.47v (National Institutes of Health, Bethesda, MD, USA) (Supplementary Figure S1A). 25 independent fields were analyzed individually for each case and the data was expressed as mean values for all patients.

### 4.8. RNA Extraction, Library Preparation and RNA Sequencing

RNA was extracted using the Universal RNA-Seq System. KAPA HyperPrep Kit was used for further depletion of ribosomal RNA and library preparation. Different adaptors were used for multiplexing samples in one sequencing run. Library concentrations and quality were measured using Qubit ds DNA HS Assay Kit (Life Technologies) and Agilent Tapestation (Agilent, Santa Clara, CA, USA). RNA sequencing was performed using Illumina HiSeq 3000 equipment (Illumina, Inc., San Diego, CA, USA) for single-end sequencing, 50 bp read length, for approximately 30 million raw reads per sample at Oncobox, LLC (Moscow, Russia), according to [44]. A data quality check was conducted using Illumina SAV. De-multiplexing was performed using Illumina Bcl2fastq2 v 2.17 software.

### 4.9. Processing and Functional Annotation of RNA Sequencing Data

RNA sequencing FASTQ files were processed with the STAR aligner [45] in ‘GeneCounts’ mode with the Ensembl human transcriptome annotation (Build version GRCh38 and transcript annotation GRCh38.89). Ensembl gene IDs were converted to HGNC gene symbols using the Complete HGNC dataset (https://www.genenames.org, database version of 23 July 2022). In total, read count values were established for 36596 annotated genes with the corresponding HGNC identifiers.

All samples analyzed in this study had a total number of uniquely aligned reads of more than 3.5 million, which was set as an internal quality control threshold established for the current RNA sequencing protocol according to [45], Table 3. Read counts were normalized using the DESeq2 R package v.1.38.0 [46]. Differential expression analysis was done using the DESeq2 package and visualized with the EnhancedVolcano R package v.1.16.0 [47]. Genes that were considered statistically significantly differentially expressed had to pass a threshold of FDR-adjusted p-values < 0.1 [48]. Gene Ontology (GO) enrichment analysis was performed using the clusterProfiler (v.4.2.1) and org.Hs.eg.db (v.3.8.2) R packages. Principal component analysis (PCA) and visualization were done for log10-transformed counts of all genes using the pca2d and prcomp R functions. Pathway activation levels were calculated for 3044 molecular pathways using Oncobox software and database [13,49].

### 4.10. Quantitative Real-Time PCR

RNA isolation from cells and tissues was performed using a total RNA isolation kit (Biolabmix, Moscow, Russia) according to the company’s protocol. Reverse transcription was performed with 1 μg DNAse-treated RNA using 5X RT MasMIX-30100 reagent mix (Dialat, Moscow, Russia). Quantitative real-time polymerase chain reaction (qRT-PCR) was performed using 5X qPCRmix-HS SYBR reagent mix (Evrogen, Moscow, Russia). Amplification was performed on the CFX96 Touch real-time PCR detection system (Bio-Rad, USA). Amplification conditions were the same for all genes studied. The initial denaturation was 3 min at 95 °C, followed by 40 cycles: denaturation at 95 °C for 15 s, annealing at 60 °C for 15 s and extension at 72 °C for 30 s. The sequences of the primers for the genes studied are listed in Supplementary Table S1. All experiments were performed in triplicate. Gene expression was normalized to the expression of human β-actin (ACTB), which was used as an internal control. CFX Manager v. 3.1 software was used for threshold cycle (Ct) calculation and data analysis. Relative mRNA expression was determined using the 2^−ΔΔCt^ method [50].

### 4.11. Statistical Tests

Statistical tests were performed using R. Statistically significant differentially expressed genes in the comparison of differentiated and non-differentiated cells were obtained using DESeq2, with an adjusted *p*-value < 0.1. To test randomness for observed numbers of overlapped differential genes or pathways between the intersecting datasets, for every comparison, 1000 random intersections were performed according to [51]. In every case, two random samples from the corresponding gene sets under comparison were taken. *p*-value of intersection significance was calculated as the expected fraction of random intersections that is equal to or higher than the experimentally observed number of overlapping genes, GO terms, or molecular pathways, depending on the items under analysis.

## 5. Conclusions

The ability of human alveolar mucosa AMC cells to differentiate in a myogenic direction has been investigated at the level of RNA gene expression. This capacity is associated with the presence of an undifferentiated subpopulation of proliferation-competent cells that are capable of producing multinucleated myotubes. AMCs showed superior characteristics to those of skeletal muscle-derived stromal cells (SMCs), isolated from bone marrow and other tissues of the body. AMCs had greater proliferative potential and were easily accessible; moreover, tissue sampling was minimally traumatic, and the resulting wound healed rapidly without requiring further rehabilitation. These properties make this type of mesenchymal stromal cell an attractive candidate for the treatment of myodegenerative diseases [52,53,54]. Here we investigated the differentiation of AMC cells in 2D and 3D culture conditions and identified core genes and molecular pathways altered during differentiation and compared these changes with those observed in differentiating SMCs. We found that AMC differentiation in 3D differed markedly from that observed under 2D conditions. Moreover, differentiation of AMCs in 2D was closer to differentiation of SMCs in 2D than to AMCs in 3D. Our results may be considered as a starting point in the search for optimal culturing conditions for effective myogenic differentiation of AMCs for subsequent scaling in biomedical production.

## Figures and Tables

**Table 1:** Statistics of samples that have passed quality control for each cell treatment type.

Cell Type	Number of Samples
2D AMC differentiated	6
2D AMC non-differentiated	5
2D SMC differentiated	7
2D SMC non-differentiated	6
3D AMC differentiated	3
3D AMC non-differentiated	5
3D SMC differentiated	2
3D SMC non-differentiated	4

**Table 2:** Top 5 up- and downregulated genes in differentiated AMCs in 2D culturing conditions.

Gene ID	Gene Full Name	log2(Fold Change)	Adjusted *p*-Value	Differential Regulation
*UNC5D*	Unc-5 Netrin Receptor D	5.64	0.069	up
*LRRC7*	Leucine-Rich Repeat-Containing Protein 7	5.29	0.078	up
*H19*	H19 Imprinted Maternally Expressed Transcript	4.48	0.048	up
*LINC01121*	Long Intergenic Non-Protein Coding RNA 1121	4.4	0.09	up
*RGS5*	Regulator of G-Protein Signaling 5	4.36	0.001	up
*HIST1H3G*	Histone Cluster 1 H3 Family Member G	−6.34	4.66 × 10^−5^	down
*CDC20*	Cell Division Cycle 20	−5.93	1.42 × 10^−7^	down
*HIST1H2AJ*	Histone Cluster 1 H2 Family Member AJ	−5.64	8.60 × 10^−8^	down
*HIST1H3B*	Histone Cluster 1 H3 Family Member B	−5.46	1.42 × 10^−7^	down
*NEK2*	NIMA (Never In Mitosis Gene A)-Related Kinase 2	−5.37	0.012	down

**Table 3:** Top up- and downregulated genes in differentiated AMCs in 3D culturing conditions.

Gene ID	Gene Full Name	log2(Fold Change)	Adjusted *p*-Value	Differential Regulation
*PLEKHA6*	Pleckstrin Homology Domain-Containing Family A Member 6	1.53	0.0647	up
*MT3*	Metallothionein 3	−8.64	0.099	down
*TUBB2B*	Tubulin Beta 2B Class Iib	−6.84	0.0442	down
*DLK1*	Delta-Like Non-Canonical Notch Ligand 1	−6.41	0.0863	down
*ISM2*	Isthmin 2	−5.83	0.0058	down
*RASGEF1A*	RasGEF Domain Family Member 1A	−5.65	0.074	down

**Table 4:** Top up- and downregulated genes in differentiated SMCs in 2D culture conditions.

Gene ID	Gene Full Name	log2(Fold Change)	Adjusted *p*-Value	Differential Regulation
*ADGRF5*	Adhesion G Protein-Coupled Receptor F5	5.68	0.0103	up
*AOC1*	Amine Oxidase Copper-Containing 1	5.24	0.0622	up
*DMRT3*	Doublesex and Mab-3 Related Transcription Factor 3	5.1	0.0768	up
*PCDH17*	Protocadherin 17	4.97	0.0103	up
*SLC47A2*	Solute Carrier Family 47 Member 2	3.72	0.0002	up
*LBP*	Lipopolysaccharide Binding Protein	−4.65	0.023	down
*RN7SL518P*	RNA, 7SL, Cytoplasmic 518 Pseudogene	−3.83	0.0982	down
*RN7SL253P*	RNA, 7SL, Cytoplasmic 253 Pseudogene	−3.03	0.0622	down
*PENK*	Proenkephalin	−2.69	0.0512	down
*RN7SL278P*	RNA, 7SL, Cytoplasmic 278 Pseudogene	−2.66	0.0917	down

**Table 5:** Top up- and downregulated genes in differentiated SMCs in 3D culturing conditions.

Gene ID	Gene Full Name	log2(Fold Change)	Adjusted *p*-Value	Differential Regulation
*CCKAR*	Cholecystokinin A Receptor	8.44	0.0006	up
*IQGAP3*	IQ Motif Containing GTPase Activating Protein 3	7.09	2.44 × 10^−6^	up
*RASGRF2*	Ras Protein-Specific Guanine Nucleotide-Releasing Factor 2	6.67	0.003	up
*ZNF536*	Zinc Finger Protein 536	6.62	0.0401	up
*OCLN*	Occludin	6.46	0.0502	up
*TMEM176A*	Transmembrane Protein 176A	−9.12	0.0002	down
*HLA-DRB1*	Major Histocompatibility Complex, Class II, DR Beta 1	−9.11	0.0005	down
*GNLY*	Granulysin	−8.36	0.0021	down
*LPL*	Lipoprotein Lipase	−8.34	0.0022	down
*CADM2*	Cell Adhesion Molecule 2	−7.92	0.0023	down

## Data Availability

The original contributions presented in the study are included in the article/Supplementary Materials. Further inquiries can be directed to the corresponding authors.

## Supplementary Materials

The following supporting information can be downloaded at https://www.mdpi.com/article/10.3390/ijms27198712/s1.
