# Supplementary material for: Molecular Mechanisms Implicated in Myogenic Differentiation of Human Alveolar Mucosa-Derived Cells
Source: Int J Mol Sci. 2026 Sep 29;27(19):8712. doi: 10.3390/ijms27198712 (PMC13665378; doi:10.3390/ijms27198712)
Supplement: Supplementary file 1 [file ijms-27-08712-s001.zip › Supplementary Table S1.pdf]

Supplementary Table S1. Sequences of the primers used

Primer name	Sequences 5'-3'
MYOG_F	GCCATCCAGTACATCGAGCG
MYOG_R	GGTTGTGGGCATCTGTAGGG
MYOD1_F	GCCACAACGGACGACTTCTA
MYOD1_R	AGTGCTCTTCGGGTTTCAGG
PAX7_F	TAGTGAGTTCGATTAGCCGC
PAX7_R	CTTCAGTGGGAGGTCAGGTTC
ACTA1_F	TTTATGAGGGCTACGCGCTG
ACTA1_R	CGCCATCTCGTTCTCGAAGT
MYH1_F	TAAGGTCGCATCTCTACGCC
MYH1_R	AAGGCTTGTTCTGGGCTTCA
ACTB_F	ATGTGGCCGAGGACTTTGATT
ACTB_F	AGTGGGGTGGCTTTTAGGATG
